# Supplementary material for: An Exploration of the Effect of the Kleier Model and Carrier-Mediated Theory to Design Phloem-Mobile Pesticides Based on Researching the N-Alkylated Derivatives of Phenazine-1-Carboxylic Acid-Glycine
Source: Molecules. 2022 Aug 6;27(15):4999. doi: 10.3390/molecules27154999 (PMC9370529; doi:10.3390/molecules27154999)
Supplement: Supplementary file 1 [file molecules-27-04999-s001.zip › molecules-1810484-supplementary.pdf]

An Exploration of the Effect of the Kleier Model and Carrier-Mediated Theory to Design Phloem-Mobile Pesticides Based on Researching the N-Alkylated Derivatives of Phenazine-1-Carboxylic Acid-Glycine

Jinlong Cai[a,1†], Yongtong Xiong[a,1†], Xiang Zhu[a], Jinyu Hu[a], Yunping Wang[a], Junkai Li[a,b] , Jianfeng Wu[c], Qinglai Wu[a, b,*]

[a]*School of Agriculture, Yangtze University, Jingmi Road 88, Jingzhou 434025, China*

[b]*Institute of Pesticides, Yangtze University, Jingmi Road 88, Jingzhou 434025, China*

[c]State Key Laboratory of Toxicology and Medical Countermeasures and Laboratory of Toxicant Analysis, Institute of Pharmacology and Toxicology, Academy of Military Medical Sciences, 27 Taiping Road, Haidian District, Beijing 100850, China;

Jinlong Cai, School of Agriculture, Yangtze University, jl2365@163.com;

Yongtong Xiong, School of Agriculture, Yangtze University, Xiongyongtong0124@163.com;

Xiang Zhu, School of Agriculture, Yangtze University, cjdxnxyzx@sina.com;

Junkai Li, School of Agriculture, Yangtze University; Institute of Pesticides, Yangtze University, junkaili@sina.com;

Jianfeng Wu, Academy of Military Medical Sciences, Institute of Pharmacology and Toxicology, ammswjf@163.com;

Qinglai Wu, School of Agriculture, Yangtze University; Institute of Pesticides, Yangtze University, wql106@163.com;

*Corresponding author. Tel/Fax: +86 716-8066314.

E-mail address: wql106@163.com (Qinglai Wu).

1

[†]These authors contributed equally to this work.

Table of Contents

Trace chromatograms of phloem sap sample **3a** ······ **Figure S1**
Trace chromatograms of phloem sap sample **3g** ······ **Figure S2**
Trace chromatograms of phloem sap sample **4a** ······ **Figure S3**
Trace chromatograms of phloem sap sample **4b** ······ **Figure S4**
Trace chromatograms of phloem sap sample **4c** ······ **Figure S5**
Trace chromatograms of phloem sap sample **4d** ······ **Figure S6**
Trace chromatograms of phloem sap sample **4e** ······ **Figure S7**
Trace chromatograms of phloem sap sample **4f** ······ **Figure S8**
Trace chromatograms of phloem sap sample **PCA-Gly** ······ **Figure S9**
Trace chromatograms of phloem sap sample **PCA** ······ **Figure S10**
^{1}H-NMR Spectrum of compound **3a** ······ **Figure S11**
HRMS Spectrum of compound **3a** ······ **Figure S12**
^{1}H-NMR Spectrum of compound **3b** ······ **Figure S13**
HRMS Spectrum of compound **3b** ······ **Figure S14**
^{1}H-NMR Spectrum of compound **3c** ······ **Figure S15**
HRMS Spectrum of compound **3c** ······ **Figure S16**
^{1}H-NMR Spectrum of compound **3d** ······ **Figure S17**
HRMS Spectrum of compound **3d** ······ **Figure S18**
^{1}H-NMR Spectrum of compound **3e** ······ **Figure S19**
HRMS Spectrum of compound **3e** ······ **Figure S20**
^{1}H-NMR Spectrum of compound **3f** ······ **Figure S21**
HRMS Spectrum of compound **3f** ······ **Figure S22**
^{1}H-NMR Spectrum of compound **3g** ······ **Figure S23**
HRMS Spectrum of compound **3g** ······ **Figure S24**
^{1}H-NMR Spectrum of compound **3h** ······ **Figure S25**
HRMS Spectrum of compound **3h** ······ **Figure S26**
^{1}H-NMR Spectrum of compound **3i** ······ **Figure S27**
HRMS Spectrum of compound **3i** ······ **Figure S28**
^{1}H-NMR Spectrum of compound **3j** ······ **Figure S29**
HRMS Spectrum of compound **3j** ······ **Figure S30**
^{1}H-NMR Spectrum of compound **3k** ······ **Figure S31**
HRMS Spectrum of compound **3k** ······ **Figure S32**
^{1}H-NMR Spectrum of compound **3l** ······ **Figure S33**
HRMS Spectrum of compound **3l** ······ **Figure S34**
^{1}H-NMR Spectrum of compound **4a** ······ **Figure S35**
HRMS Spectrum of compound **4a** ······ **Figure S36**
^{1}H-NMR Spectrum of compound **4b** ······ **Figure S37**
HRMS Spectrum of compound **4b** ······ **Figure S38**
^{1}H-NMR Spectrum of compound **4c** ······ **Figure S39**
HRMS Spectrum of compound **4c** ······ **Figure S40**
^{1}H-NMR Spectrum of compound **4d** ······ **Figure S41**
HRMS Spectrum of compound **4d** ······ **Figure S42**

^{1}H-NMR Spectrum of compound **4e**······**Figure S43**
HRMS Spectrum of compound **4e**······**Figure S44**
^{1}H-NMR Spectrum of compound **4f**······**Figure S45**
HRMS Spectrum of compound **4f**······**Figure S46**

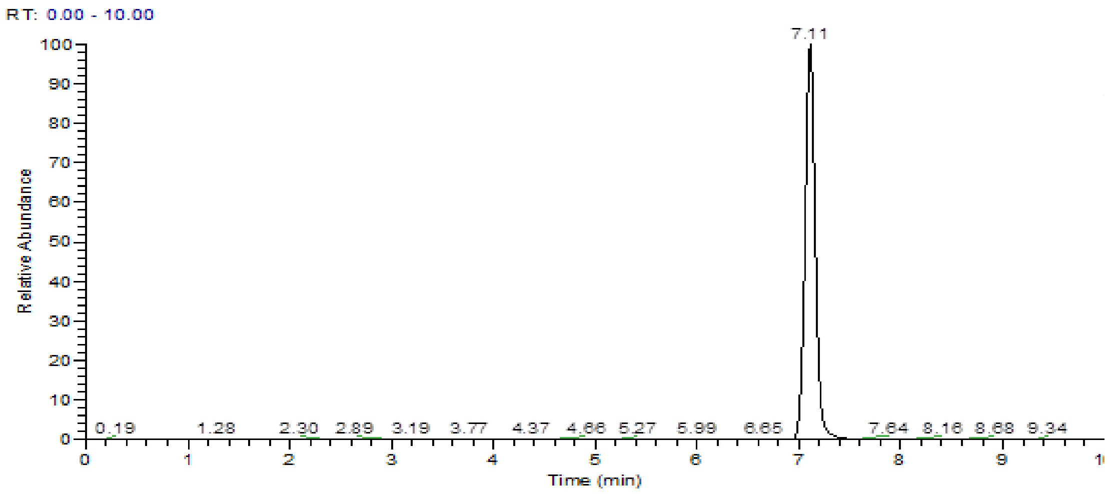

3a-A

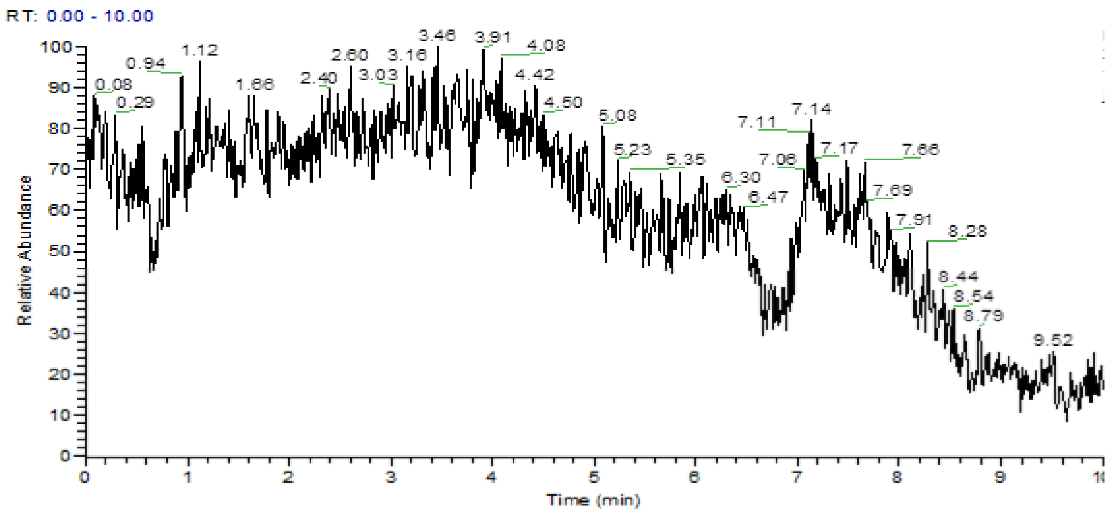

3a-B

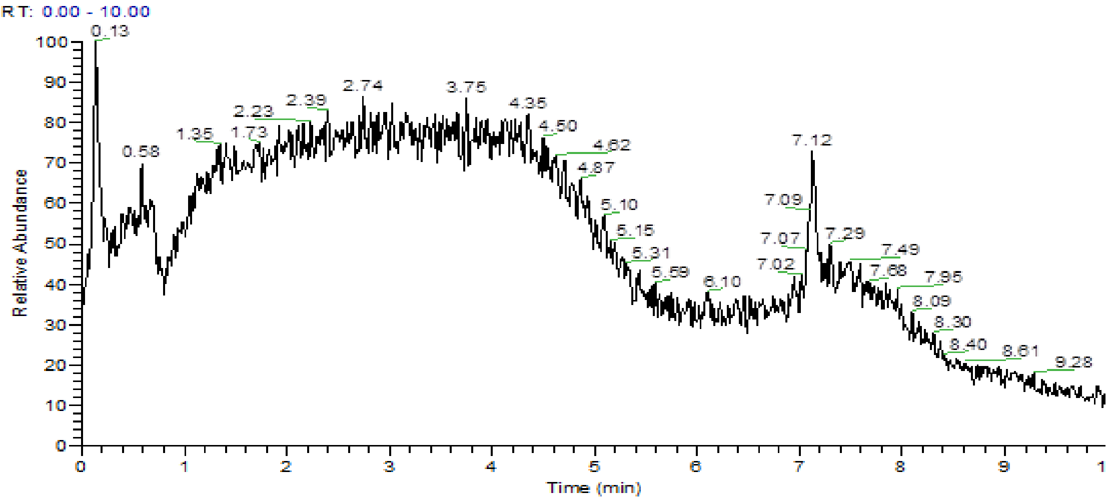

3a-C

Figure S1. *R. communis* sap analysis by UHPLC-MS. **3a-A**: standard sample of **3a** (RT: 7.11 min); **3a-B**: control, the cotyledons were incubated in the standard medium; **3a-C**: treated set, the cotyledons were incubated in the same solution with **3a** at 0.2 mM concentration.

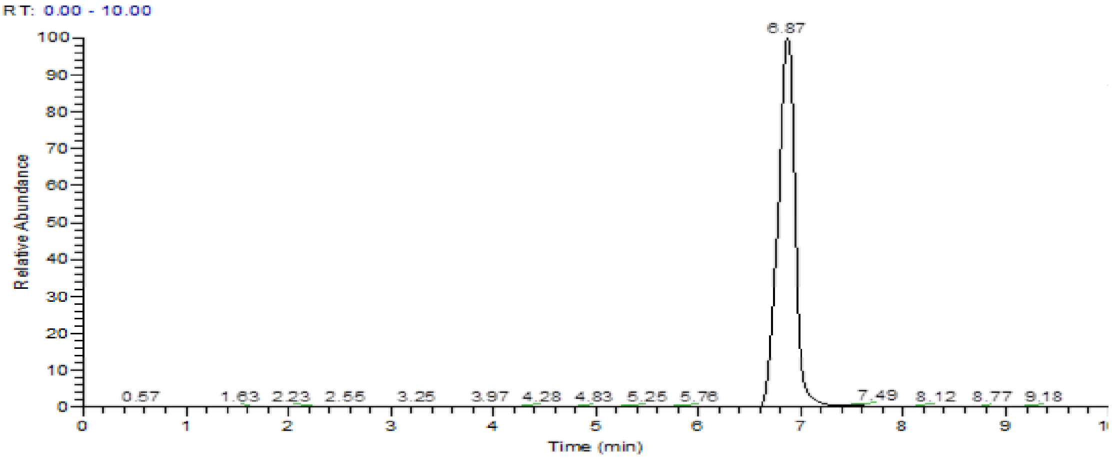

3g-A

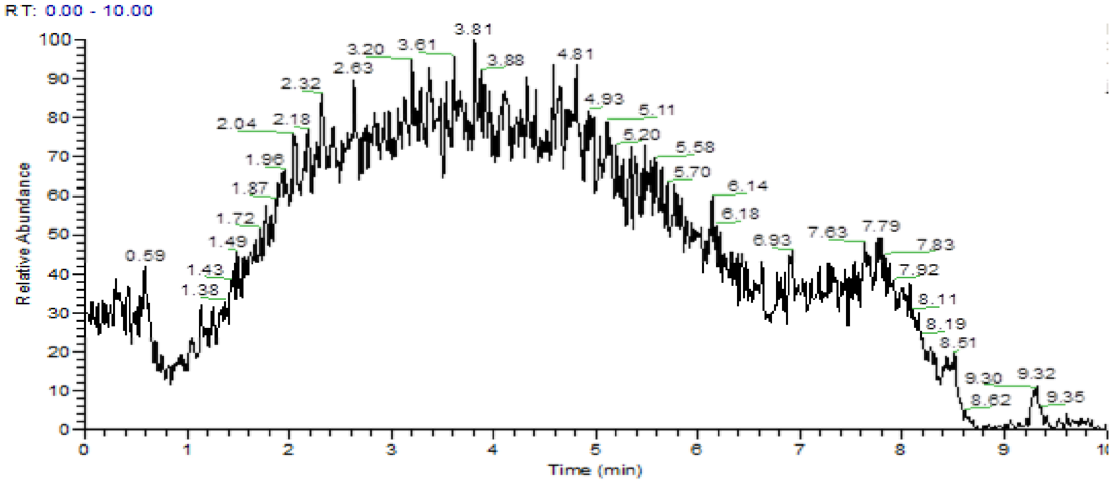

3g-B

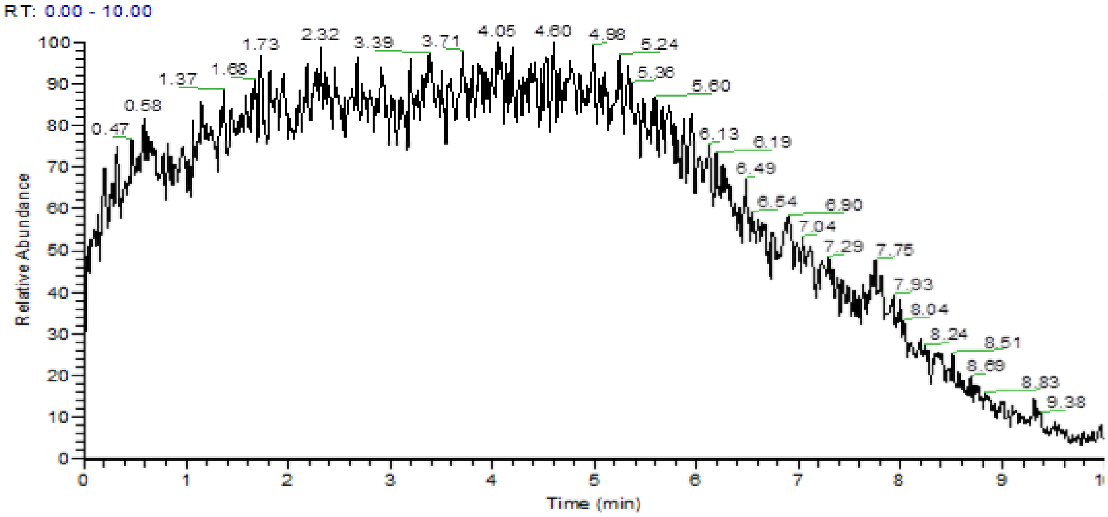

3g-C

Figure S2. *R. communis* sap analysis by UHPLC-MS. **3g-A**: standard sample of **3g** (RT: 6.87 min); **3g-B**: control, the cotyledons were incubated in the standard medium; **3g-C**: treated set, the cotyledons were incubated in the same solution with **3g** at 0.2 mM concentration.

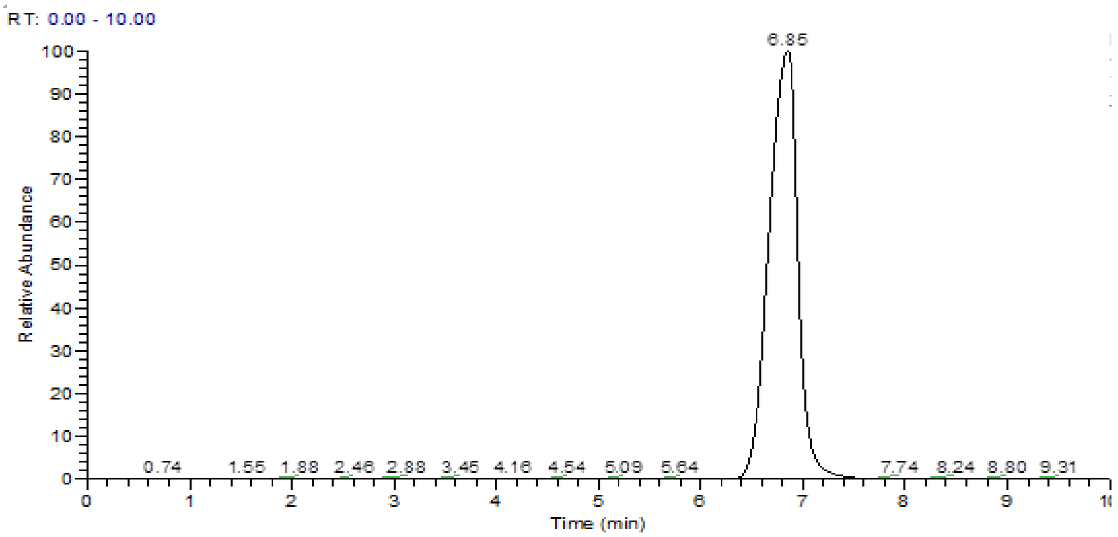

4a-A

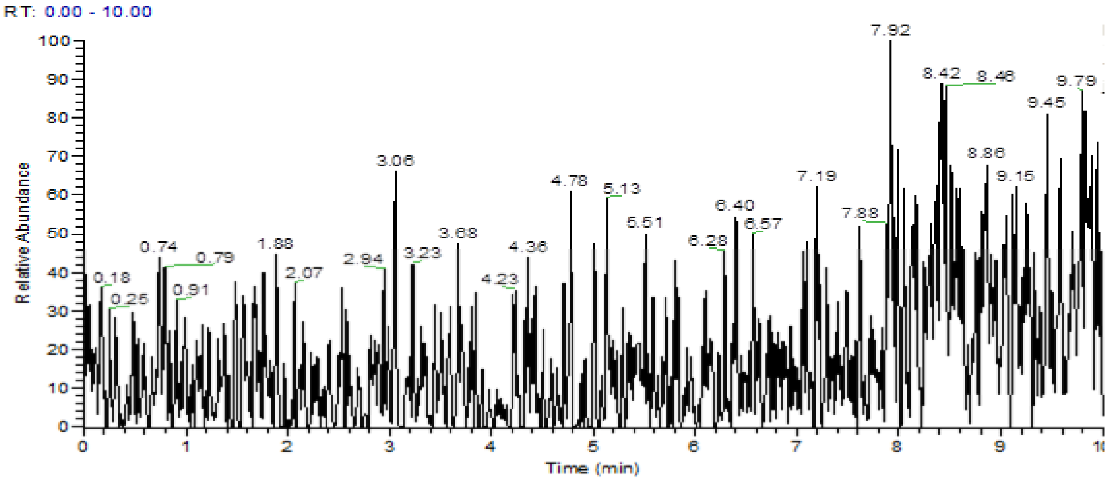

4a-B

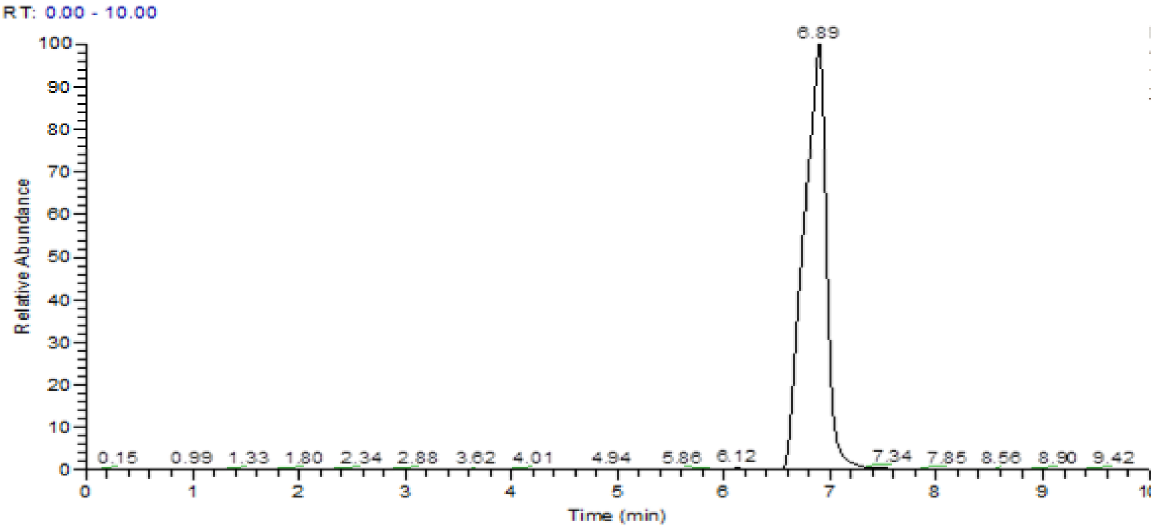

4a-C

Figure S3. *R. communis* sap analysis by UHPLC-MS. **4a-A**: standard sample of **4a** (RT: 6.85 min); **4a-B**: control, the cotyledons were incubated in the standard medium; **4a-C**: treated set, the cotyledons were incubated in the same solution with **4a** (RT: 6.89 min) at 0.2 mM concentration.

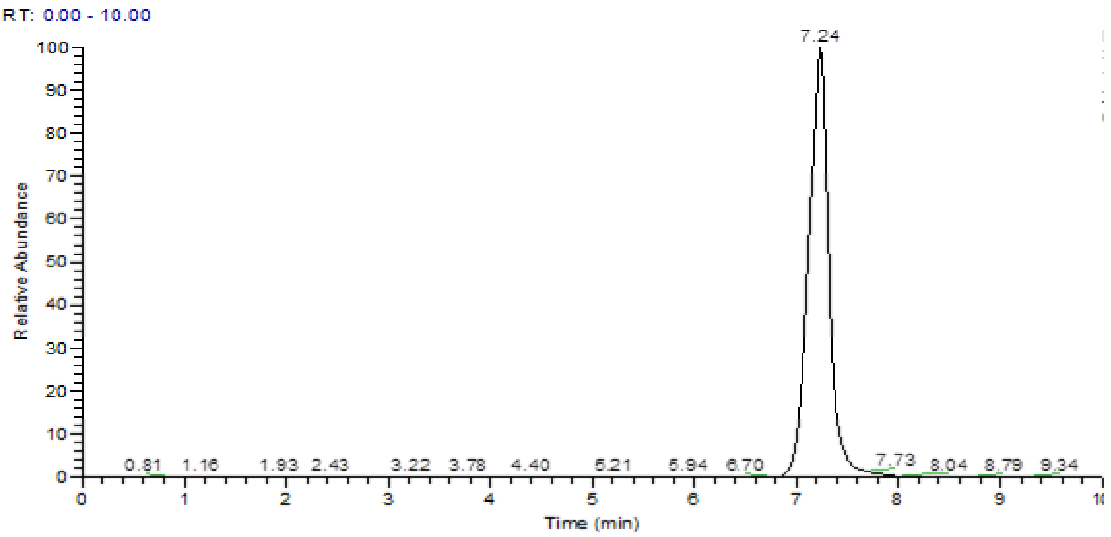

4b-A

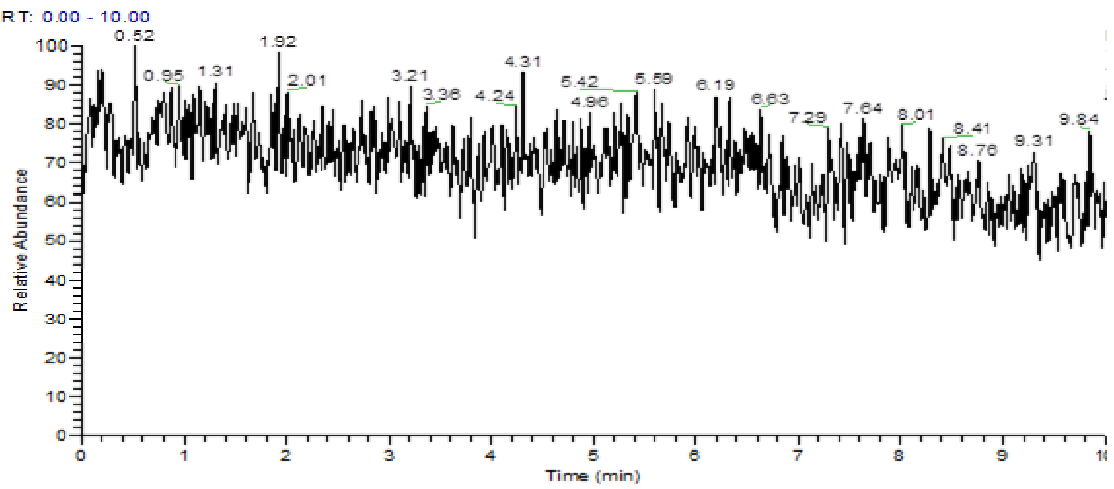

4b-B

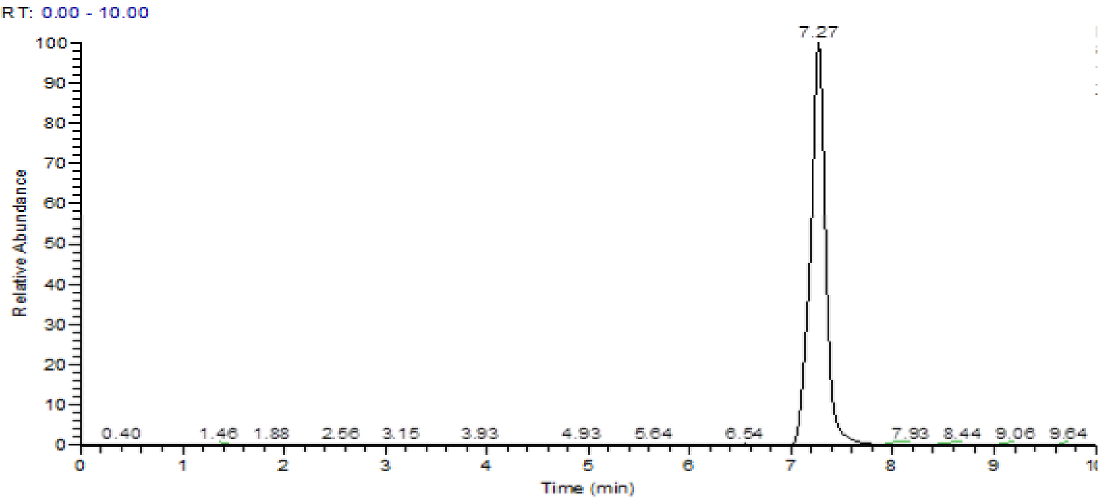

4b-C

Figure S4. *R. communis* sap analysis by UHPLC-MS. **4b-A**: standard sample of **4b** (RT: 7.24 min); **4b-B**: control, the cotyledons were incubated in the standard medium; **4b-C**: treated set, the cotyledons were incubated in the same solution with **4b** (RT: 7.27 min) at 0.2 mM concentration.

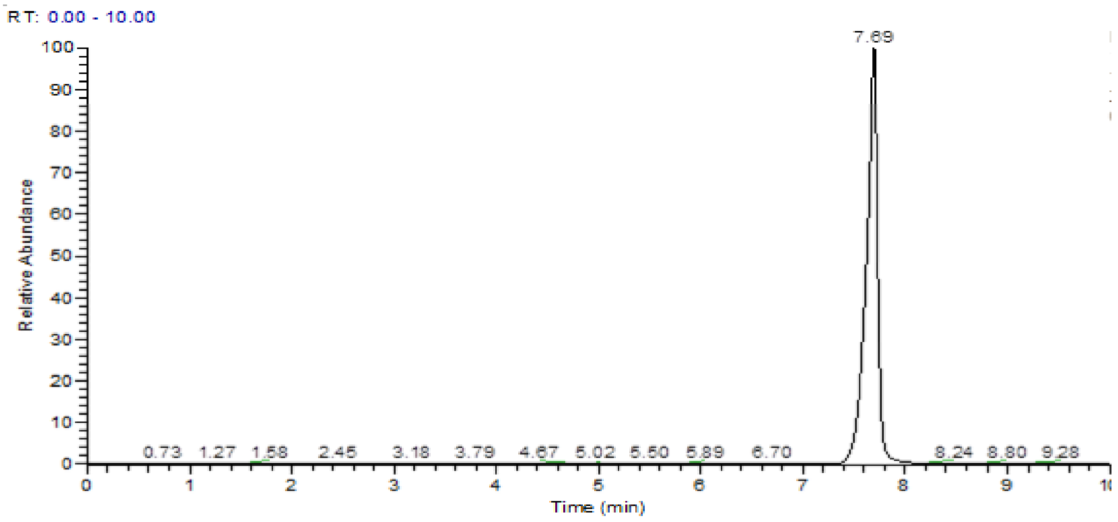

4c-A

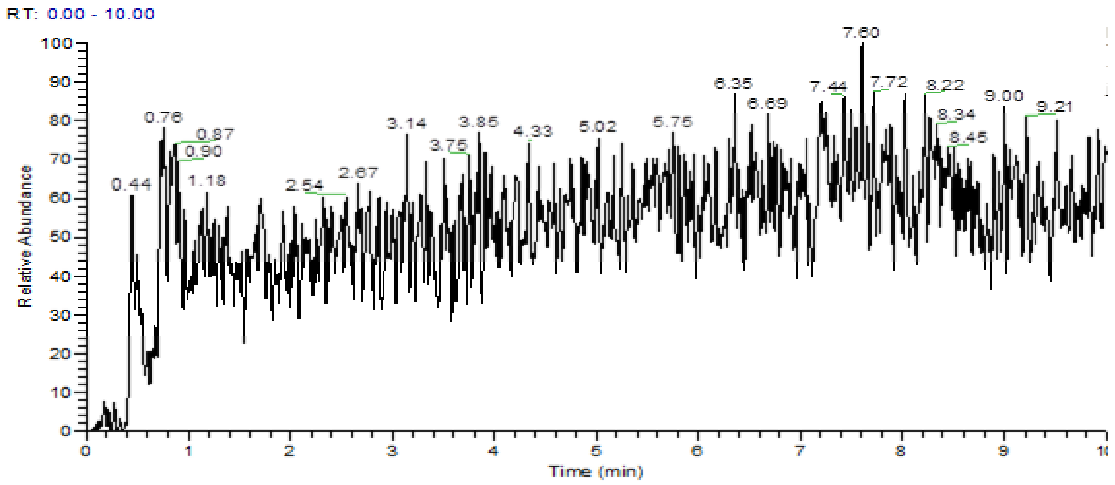

4c-B

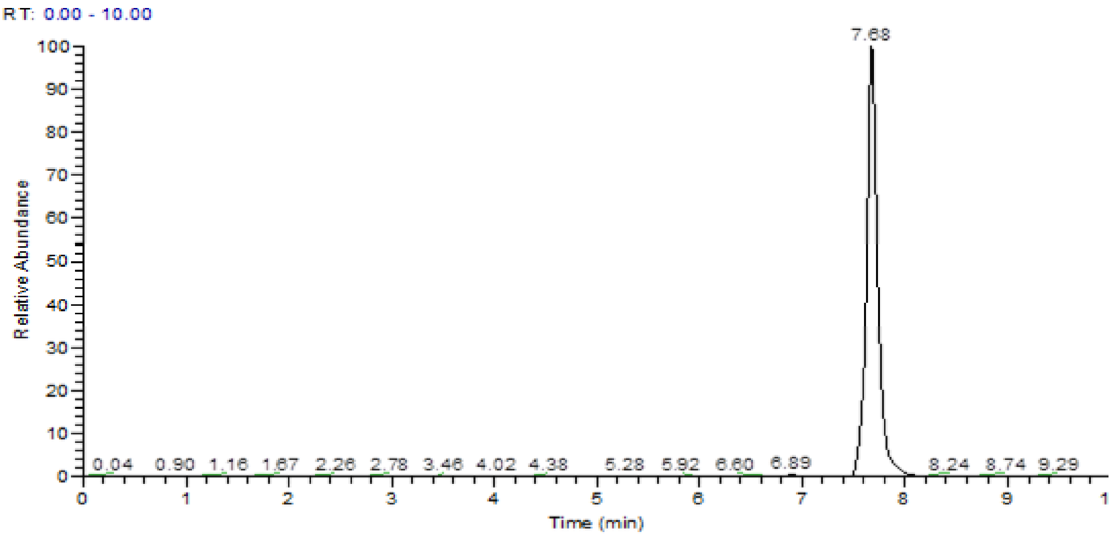

4c-C

Figure S5. *R. communis* sap analysis by UHPLC-MS. **4c-A**: standard sample of **4c** (RT: 7.69 min); **4c-B**: control, the cotyledons were incubated in the standard medium; **4c-C**: treated set, the cotyledons were incubated in the same solution with **4c** (RT: 7.68 min) at 0.2 mM concentration.

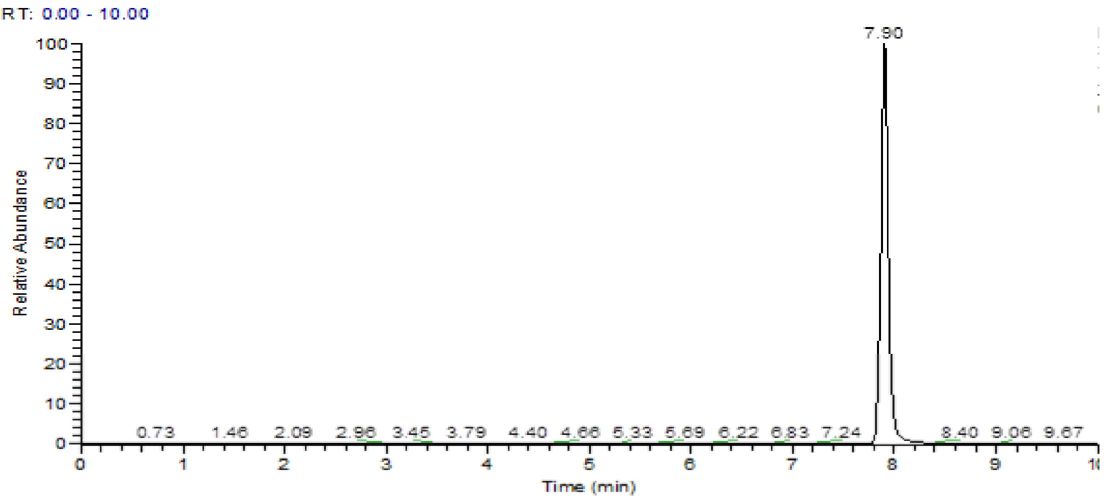

4d-A

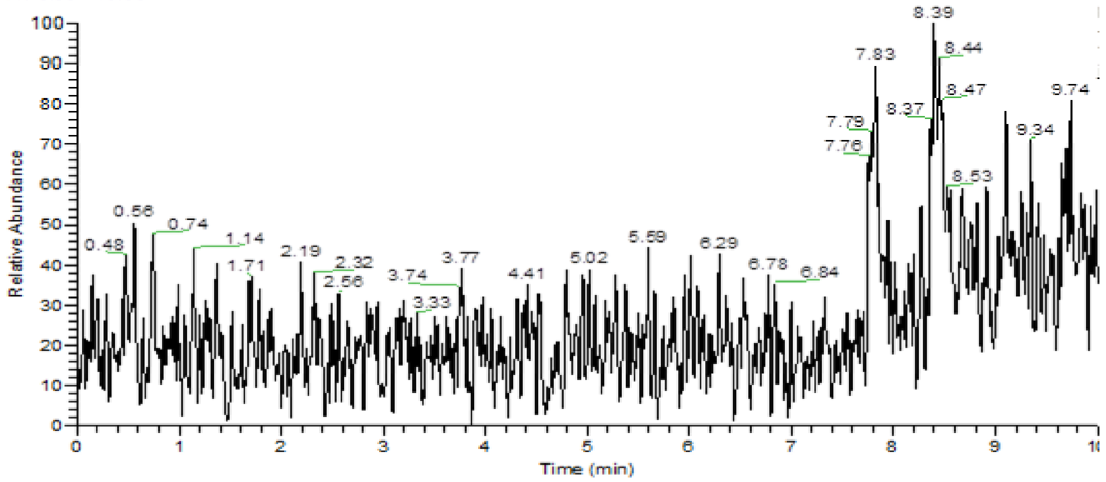

4d-B

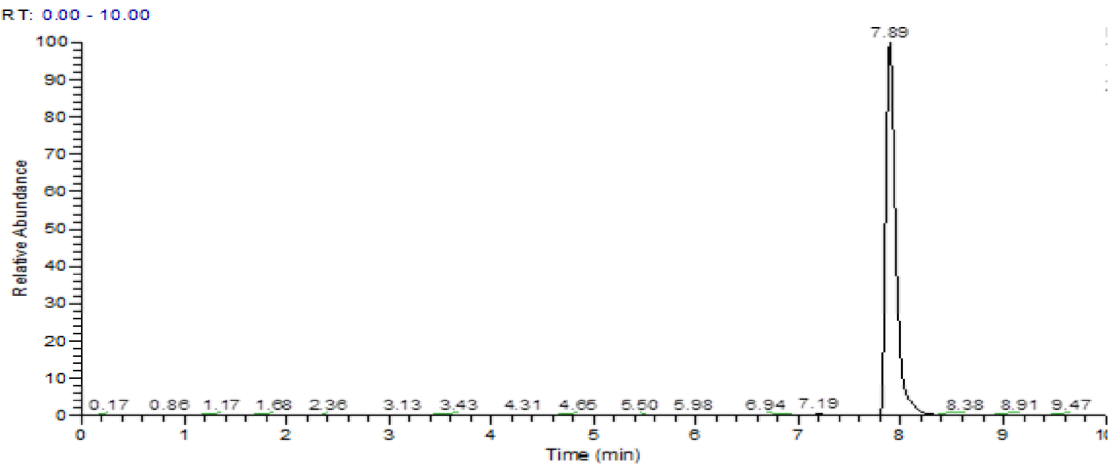

4d-C

Figure S6. *R. communis* sap analysis by UHPLC-MS. **4d-A**: standard sample of **4d** (RT: 7.90 min); **4d-B**: control, the cotyledons were incubated in the standard medium; **4d-C**: treated set, the cotyledons were incubated in the same solution with **4d** (RT: 7.89 min) at 0.2 mM concentration.

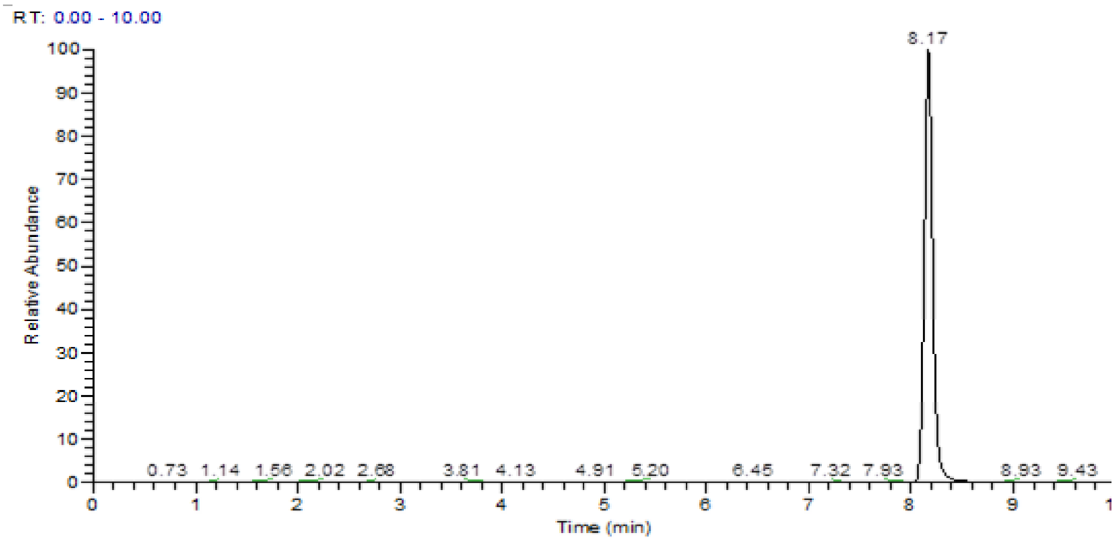

4e-A

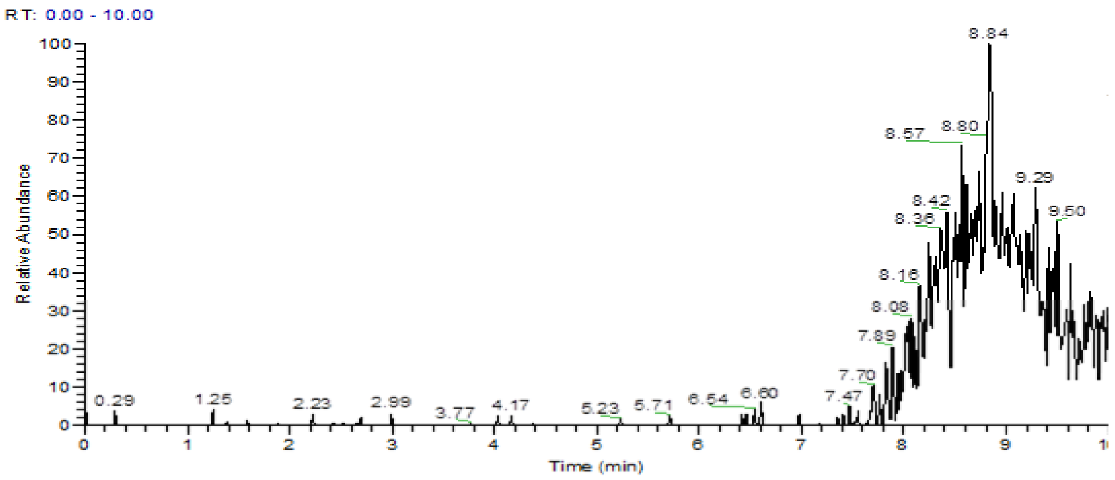

4e-B

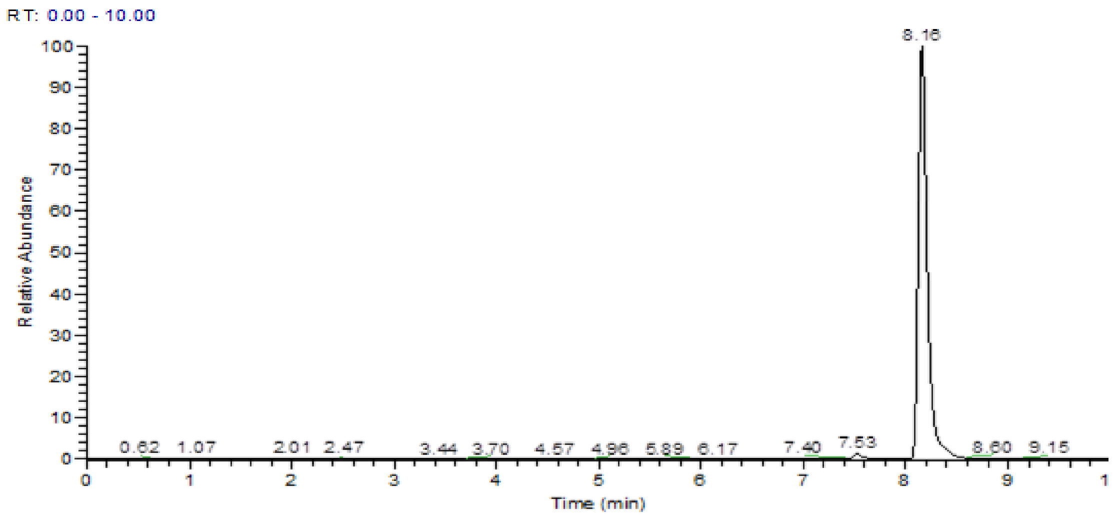

4e-C

Figure S7. *R. communis* sap analysis by UHPLC-MS. **4e-A**: standard sample of **4e** (RT: 8.17 min); **4e-B**: control, the cotyledons were incubated in the standard medium; **4e-C**: treated set, the cotyledons were incubated in the same solution with **4e** (RT: 8.16 min) at 0.2 mM concentration.

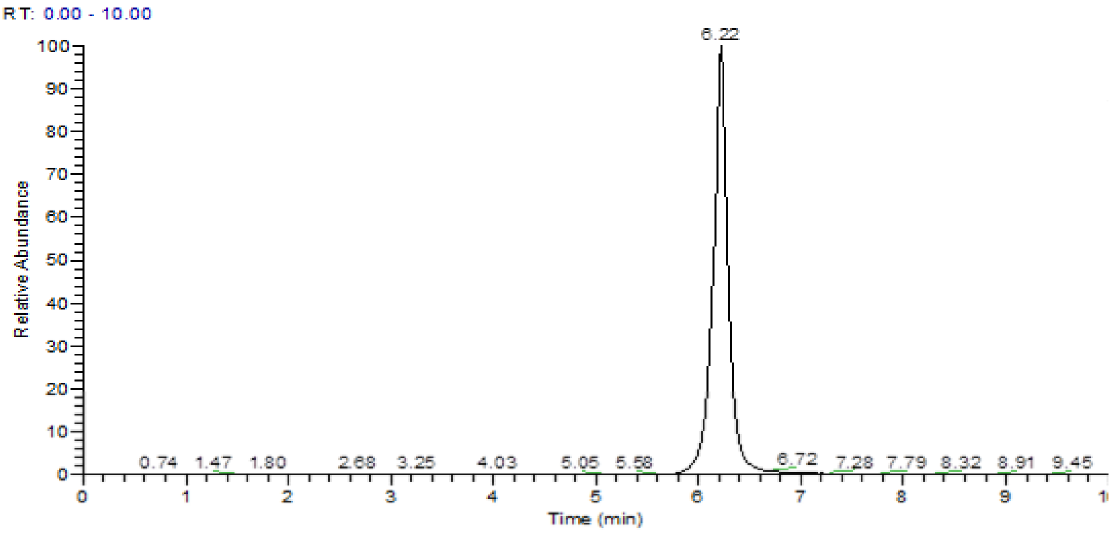

4f-A

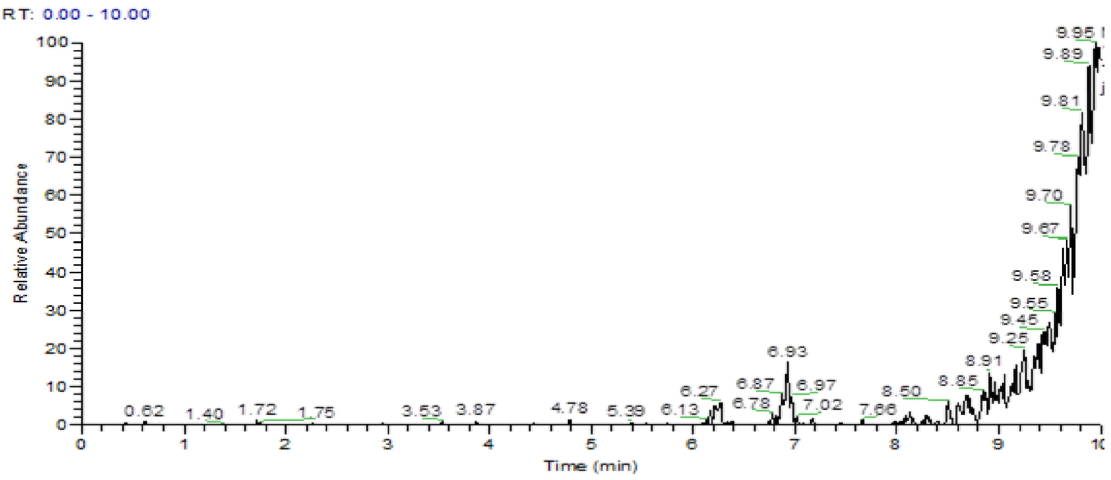

4f-B

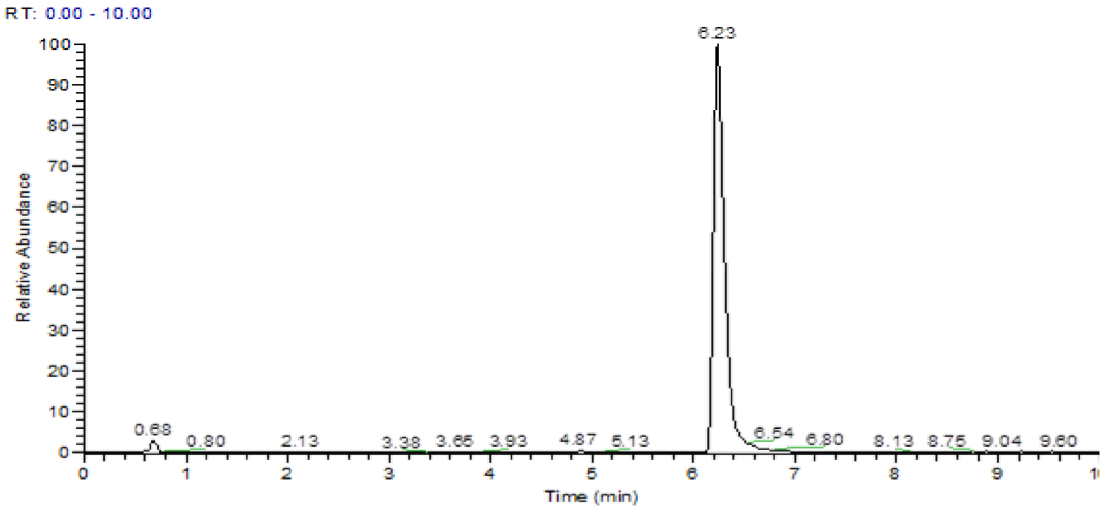

4f-C

Figure S8. *R. communis* sap analysis by UHPLC-MS. **4f-A**: standard sample of **4f** (RT: 6.22 min); **4f-B**: control, the cotyledons were incubated in the standard medium; **4f-C**: treated set, the cotyledons were incubated in the same solution with **4f** (RT: 6.23 min) at 0.2 mM concentration.

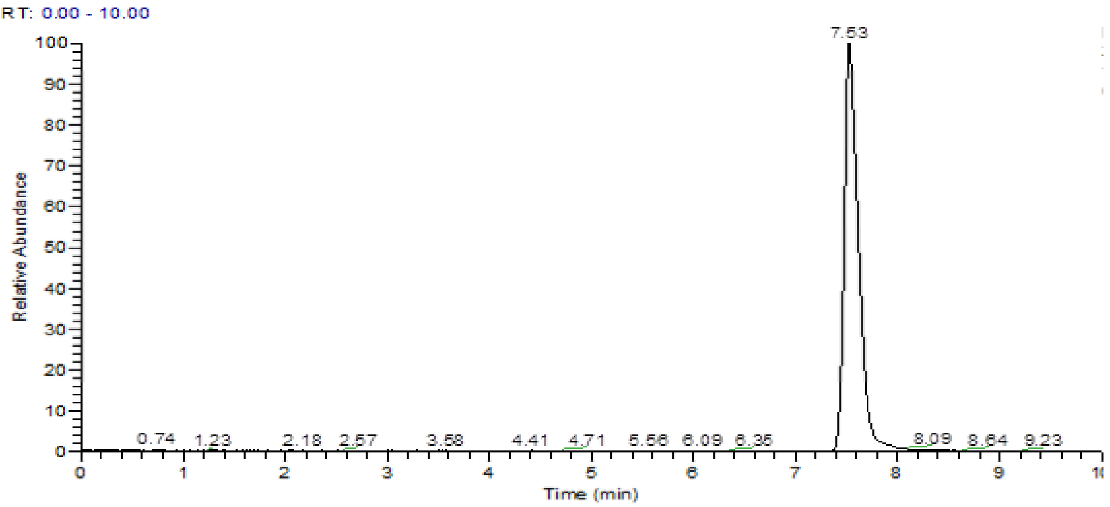

PCA-Gly-A

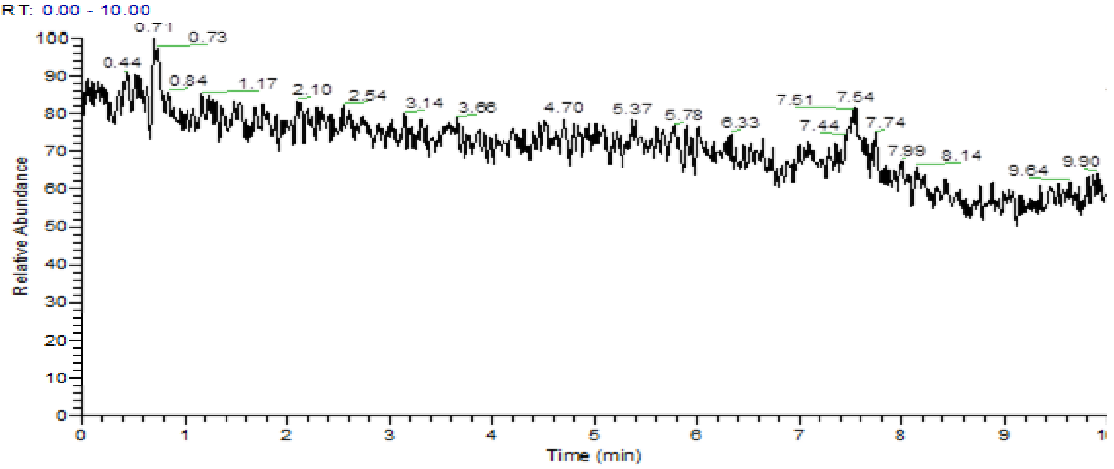

PCA-Gly-B

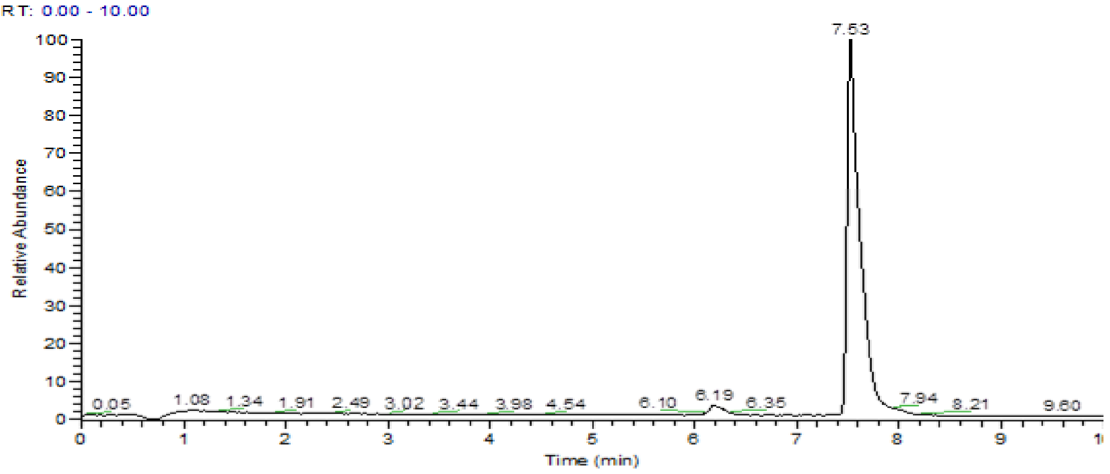

PCA-Gly-C

Figure S9. *R. communis* sap analysis by UHPLC-MS. **PCA-Gly-A**: standard sample of **PCA-Gly** (RT: 7.53 min); **PCA-Gly-B**: control, the cotyledons were incubated in the standard medium; **PCA-Gly-C**: treated set, the cotyledons were incubated in the same solution with **PCA-Gly** (RT: 7.53 min) at 0.2 mM concentration.

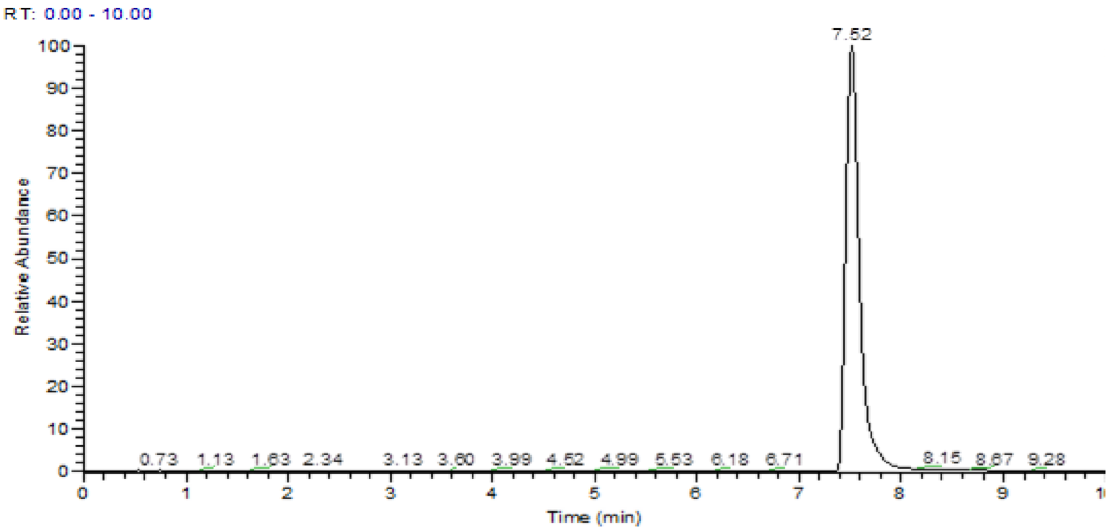

PCA-A

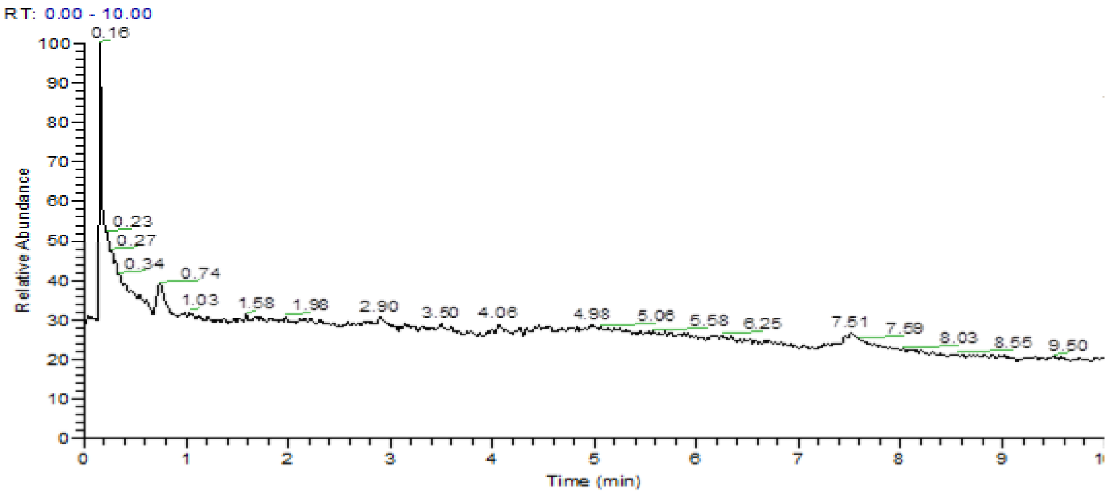

PCA-B

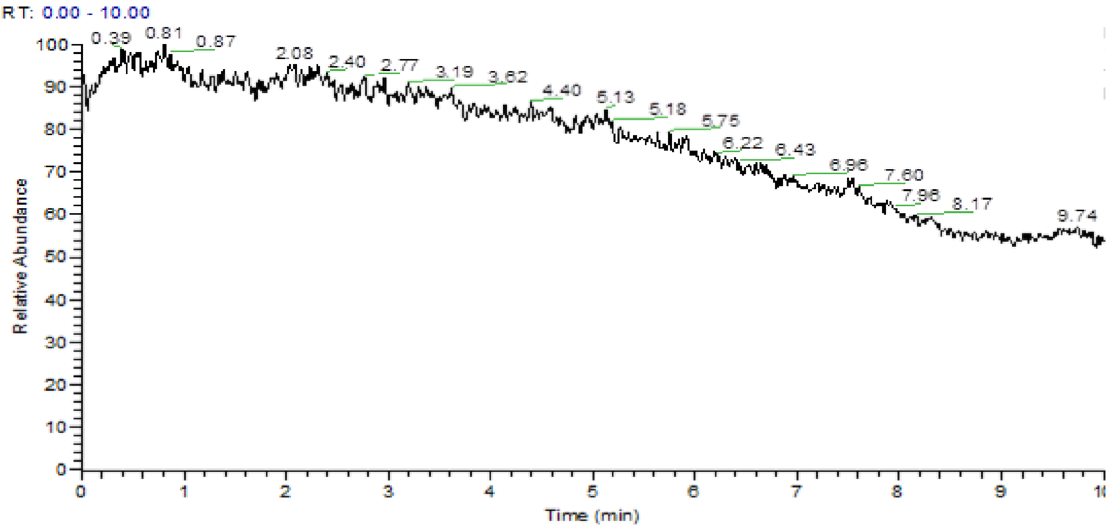

PCA-C

Figure S10. *R. communis* sap analysis by UHPLC-MS. **PCA-A**: standard sample of **PCA** (RT: 7.52 min); **PCA-B**: control, the cotyledons were incubated in the standard medium; **PCA-C**: treated set, the cotyledons were incubated in the same solution with **PCA** at 0.2 mM concentration.

Ethyl 2-(N-methylphenazine-1-carboxamido)acetate (3a) :

Yellow solid; yield: 86%; m.p. 115 – 117°C; ^{1}H NMR (400 MHz, $CDCl_3$) δ 8.33 – 8.21 (m, 3.00H, Phenazine-H), 7.94 – 7.79 (m, 4.09H, Phenazine-H), 5.00 (d, J = 16.2 Hz, 0.67H, $COOCH_2$), 4.32 (q, J = 7.2 Hz, 1.31H, N-CH_2), 4.11 – 4.00 (m, 1.34H,$COOCH_2$), 3.84 (d, J = 19.8 Hz, 0.71H, N-CH_2), 3.39 (s, 1.18H, N-CH_3), 2.89 (s, 1.95H, N-CH_3), 1.37 (t, J = 7.2 Hz, 1.94H, Methylene-CH_3), 1.11 (t, J = 7.2 Hz, 1.17H, Methylene-CH_3). HRMS calcd for $C_{18}H_{17}N_3O_3$ $[M+H]^+$: 324.1343, found 324.1337.

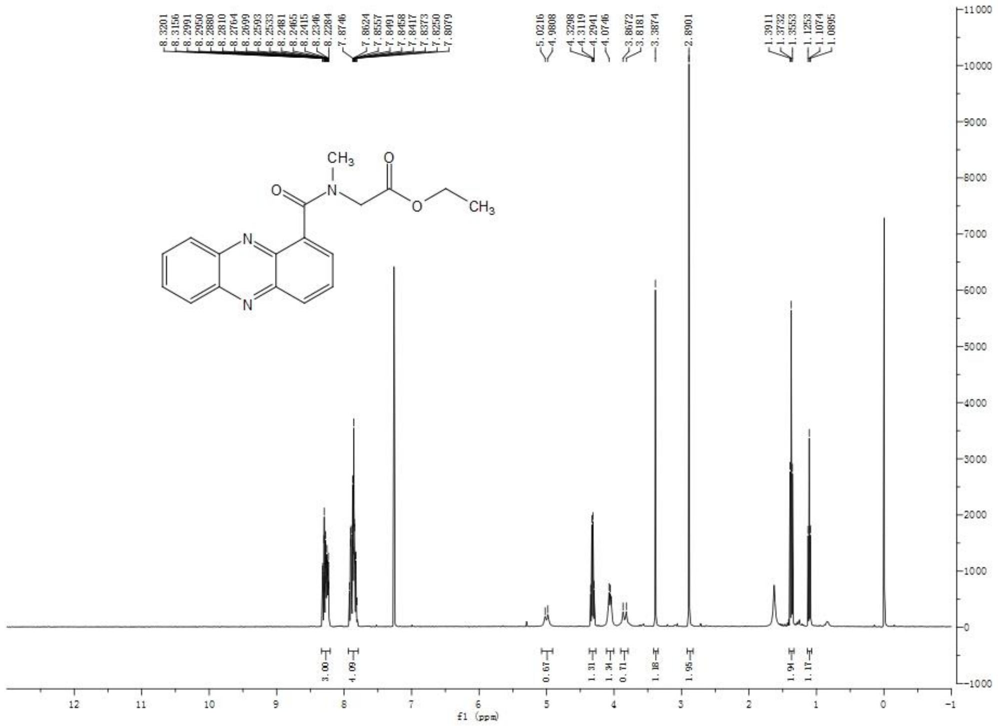

Figure S11. ^{1}H-NMR Spectrum of compound **3a**

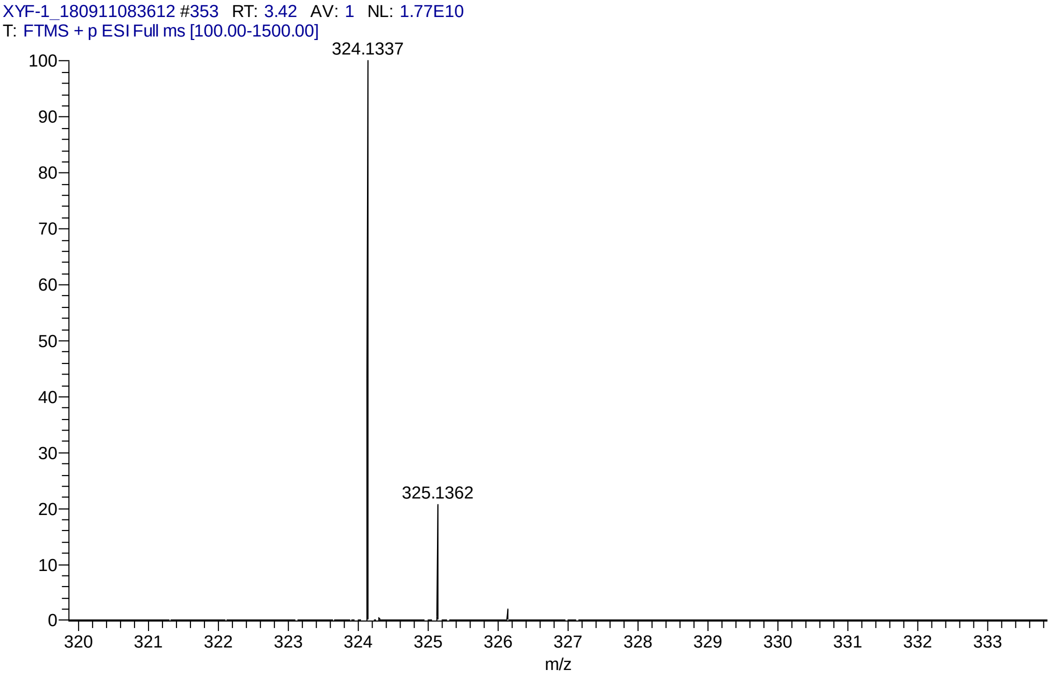

Figure S12. HRMS Spectrum of compound **3a**

Ethyl 2-(N-ethylphenazine-1-carboxamido)acetate (3b) :

Yellow solid; yield: 76%; m.p. 133 – 135°C; ^{1}H NMR (400 MHz, $CDCl_3$) δ 8.34 – 8.18 (m, 3.00H, Phenazine-H), 7.92 – 7.79 (m, 4.04H, Phenazine-H), 4.88 (d, *J* = 17.2 Hz, 0.69H, N-CH_2-Methyl), 4.33 (q, *J* = 7.2 Hz, 1.38H, N-CH_2), 4.17 – 3.98 (m, 1.72H, $COOCH_2$), 3.82 (s, 0.73H, N-CH_2), 3.65 (s, 0.38H, $COOCH_2$), 3.24 (dt, *J* = 14.8, 7.2 Hz, 1.36H, N-CH_2-Methyl), 1.40 (dt, *J* = 18.8, 7.2 Hz, 3.08H, Methylene-CH_3), 1.07 (dt, *J* = 24.2, 7.2 Hz, 3.09H, N-Methylene-CH_3). HRMS calcd for $C_{19}H_{19}N_3O_3$ $[M+H]^+$: 338.1499, found 338.1491.

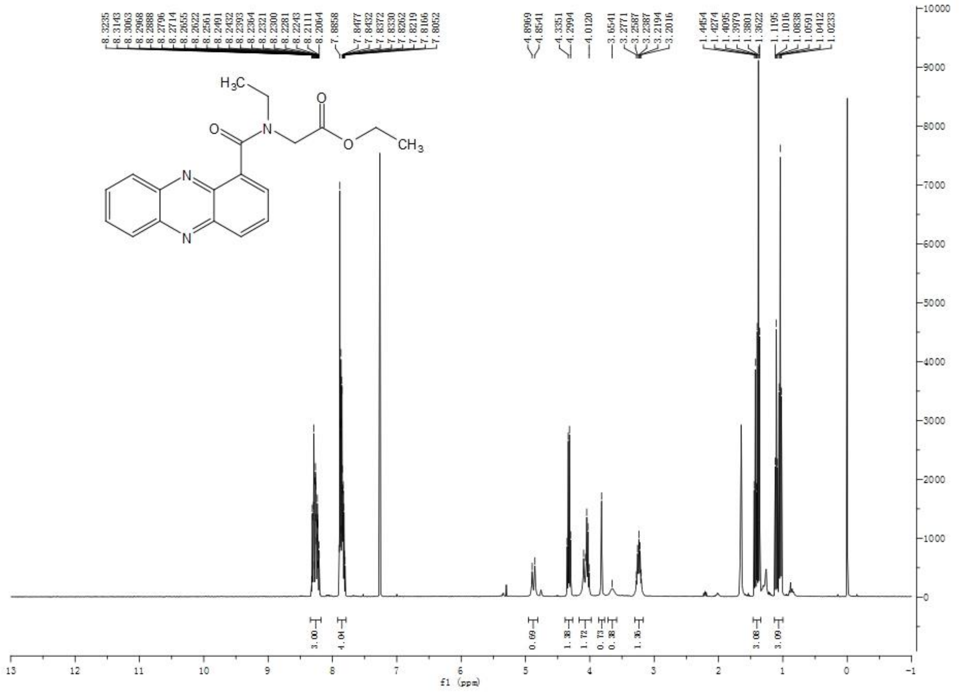

Figure S13. ^{1}H-NMR Spectrum of compound **3b**

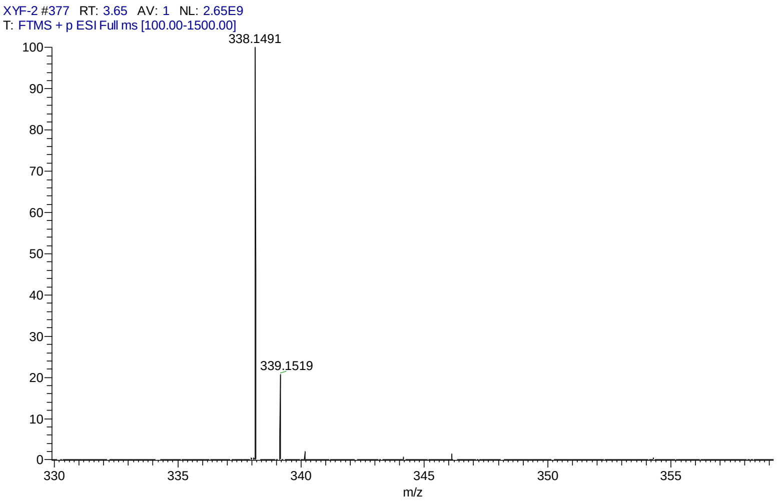

Figure S14. HRMS Spectrum of compound **3b**

Ethyl 2-(N-isopropylphenazine-1-carboxamido)acetate (3c) :

Yellow solid; yield: 73%; m.p. 131 – 132°C; ^{1}H NMR (400 MHz, $CDCl_3$) δ 8.33 – 8.22 (m, 3.09H, Phenazine-H), 7.89 – 7.82 (m, 4.05H, Phenazine-H), 4.64 (d, J = 16.8 Hz, 0.98H, N-CH), 4.33 (qd, J = 7.2, 1.8 Hz, 1.98H, $COOCH_2$), 3.96 (d, J = 16.8 Hz, 1.06H, N-CH_2), 3.77 – 3.72 (m, 1.02H, N-CH_2), 1.38 (t, J = 7.2 Hz, 2.80H, N-Methylene-CH_3), 1.15 – 1.05 (m, 6.00H, 2×CH_3). HRMS calcd for $C_{20}H_{19}N_3O_3$ $[M+H]^+$: 352.1656, found 352.1648.

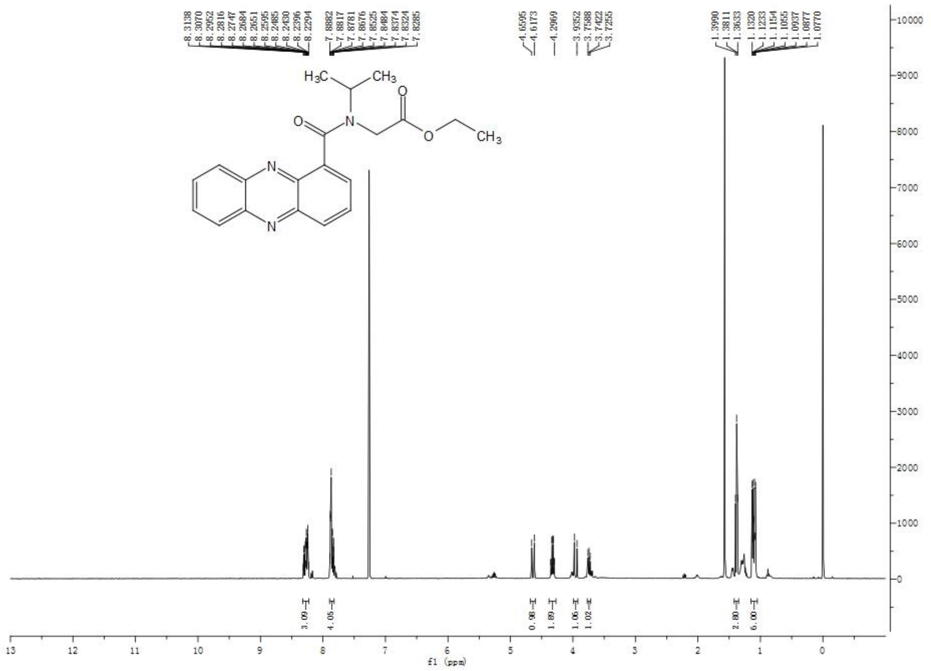

Figure S15. ^{1}H-NMR Spectrum of compound **3c**

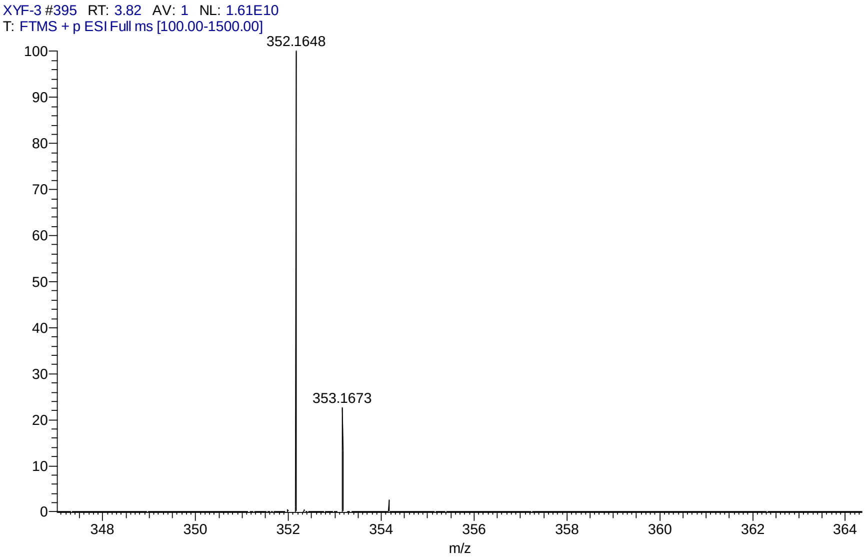

Figure S16. HRMS Spectrum of compound **3c**

Ethyl 2-(N-tert-butylphenazine-1-carboxamido)acetate (3d) :

Yellow solid; yield: 83%; m.p. 114 – 116°C; ^{1}H NMR (400 MHz, $CDCl_3$) δ 8.27 – 8.17 (m, 3.00H, Phenazine-H), 7.90 – 7.73 (m, 4.10H, Phenazine-H), 4.10 – 3.96 (m, 3.01H, $COOCH_2$, N-CH_2), 3.78 (d, J = 19.0 Hz, 1.03H, N-CH_2), 1.72 (s, 8.91H, 3×CH_3), 1.13 (t, J = 7.2 Hz, 3.02H, N-Methylene-CH_3). HRMS calcd for $C_{21}H_{23}N_3O_3$ $[M+H]^+$: 366.1812, found 366.1808.

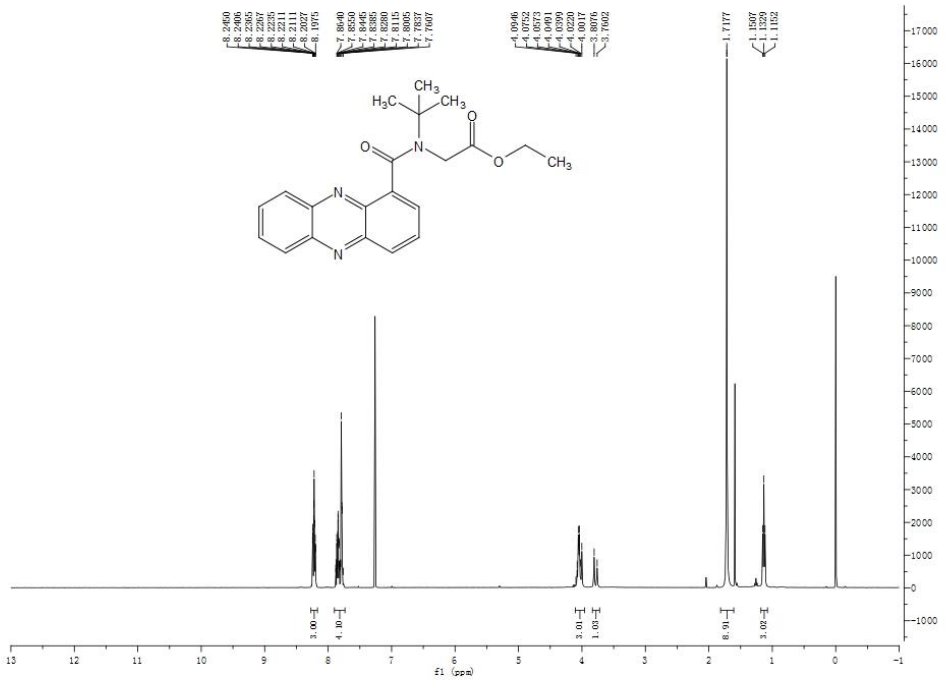

Figure S17. ^{1}H-NMR Spectrum of compound **3d**

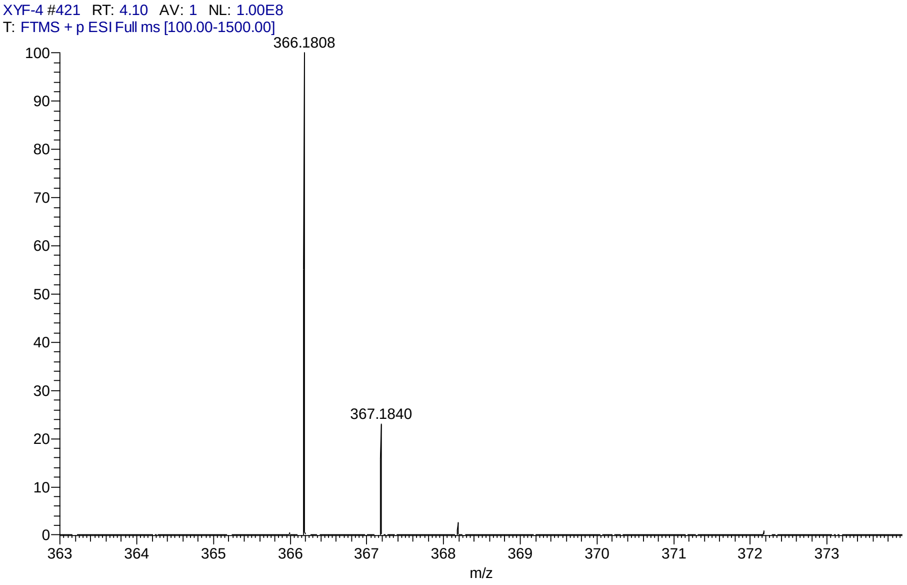

Figure S18. HRMS Spectrum of compound **3d**

Ethyl 2-(N-benzylphenazine-1-carboxamido)acetate (3e) :

Yellow solid; yield: 85%; m.p. 138 – 140°C; ^{1}H NMR (400 MHz, $CDCl_3$) δ 8.38 – 8.20 (m, 3.10H, Phenazine-H), 7.98 – 7.82 (m, 4.00H, Phenazine-H), 7.74 (d, J = 7.2 Hz, 1.00H, Benzene-H), 7.50 (dd, J = 13.2, 5.8 Hz, 1.10H, Benzene-H), 7.37 (dd, J = 17.2, 7.2 Hz, 1.60H, Benzene-H), 7.25 – 7.15 (m, 1.49H, Benzene-H), 4.82 (d, J = 17.4 Hz, 0.70H, Benzene-CH_2), 4.35 (d, J = 15.2 Hz, 0.97H, $COOCH_2$), 4.29 (q, J = 7.2 Hz, 1.24H, Benzene-CH_2), 4.03 (q, J = 7.2 Hz, 1.11H, $COOCH_2$), 3.89 (d, J = 17.2 Hz, 0.71H, N-CH_2), 3.70 (d, J = 16.8 Hz, 1.37H, N-CH_2), 1.34 (t, J = 7.2 Hz, 1.57H, N-Methylene-CH_3), 1.09 (t, J = 7.2 Hz, 1.56H, N-Methylene-CH_3). HRMS calcd for $C_{24}H_{21}N_3O_3$ $[M+H]^+$: 400.1656, found 400.1647.

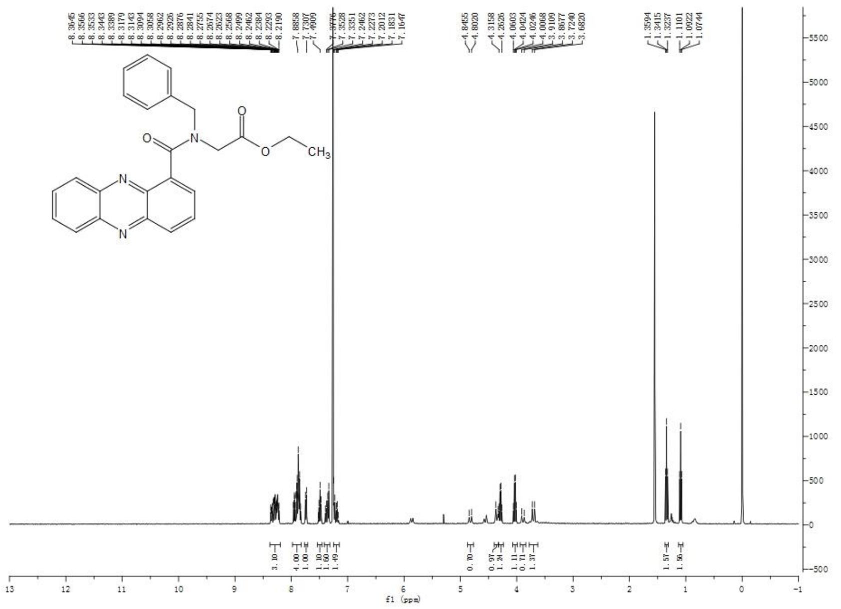

Figure S19. ^{1}H-NMR Spectrum of compound **3e**

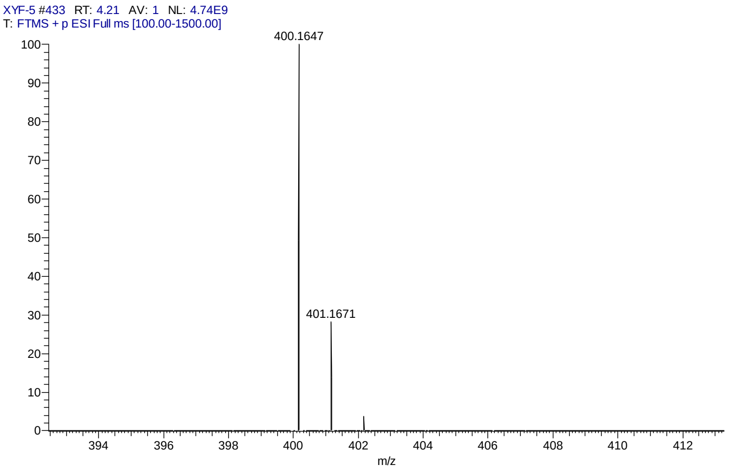

Figure S20. HRMS Spectrum of compound **3e**

Diethyl 2,2'-((phenazine-1-carbonyl)azanediyl)diacetate (3f) :

Yellow solid; yield: 89%; m.p. 95 – 97°C; ^{1}H NMR (400 MHz, $CDCl_3$) δ 8.38 – 8.19 (m, 3.00H, Phenazine-H), 7.98 – 7.79 (m, 4.02H, Phenazine-H), 4.69 (s, 2.02H, N-CH_2), 4.32 (q, J = 7.2 Hz, 2.01H, N-CH_2), 3.96 (s, 4.02H, $COOCH_2$), 1.37 (t, J = 7.2 Hz, 3.00H, CH_3), 0.97 (t, J = 7.2 Hz, 3.00H, CH_3). HRMS calcd for $C_{21}H_{21}N_3O_5$ $[M+H]^+$: 396.1554, found 396.1548.

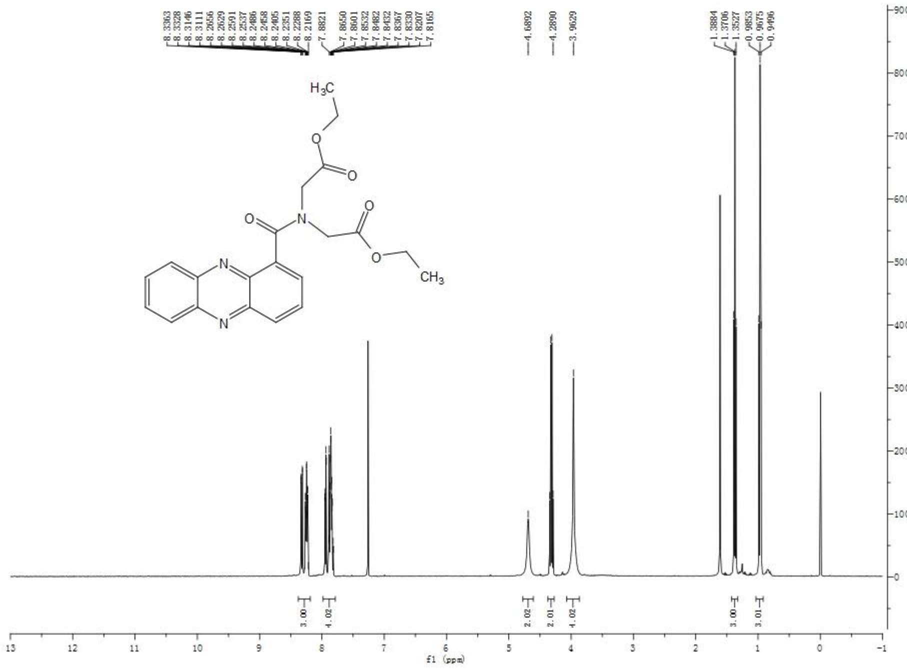

Figure S21. ^{1}H-NMR Spectrum of compound **3f**

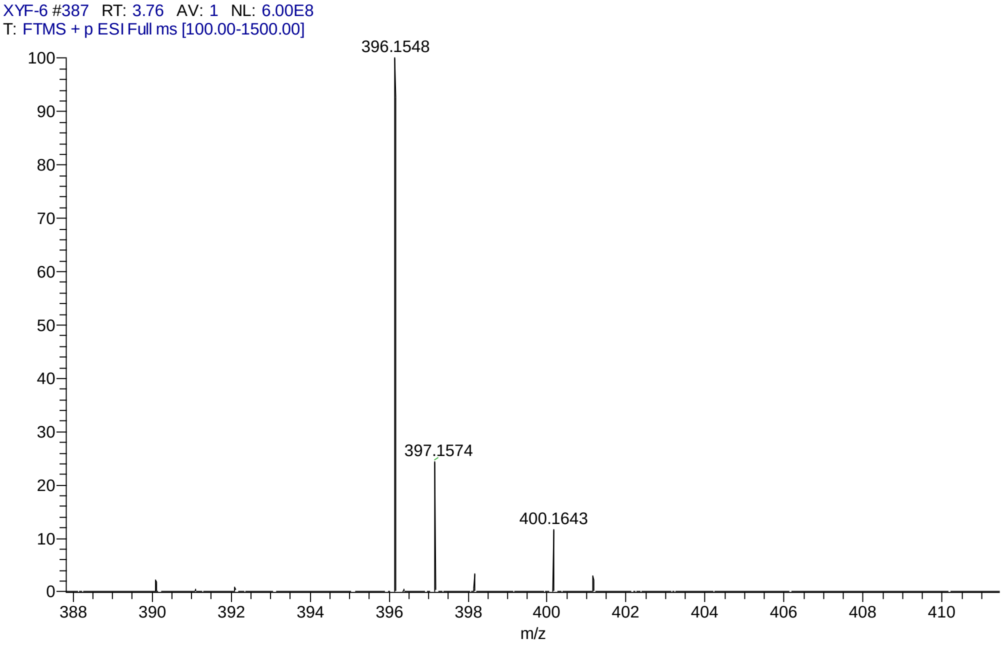

Figure S22. HRMS Spectrum of compound **3f**

Methyl 2-(N-methylphenazine-1-carboxamido)acetate (3g) :

Yellow solid; yield: 90%; m.p. 123 – 125°C; ^{1}H NMR (400 MHz, $CDCl_3$) δ 8.33 – 8.21 (m, 3.00H, Phenazine-H), 7.94 – 7.80 (m, 4.08H, Phenazine-H), 4.99 (d, *J* = 17.2 Hz, 0.68H, $COOCH_3$), 4.11 (d, *J* = 16.2 Hz, 0.82H, N-CH_2), 3.87 (s, 2.31H, $COOCH_3$), 3.60 (s, 1.12H, N-CH_2), 3.39 (s, 1.02H, N-CH_3), 2.89 (s, 2.02H, N-CH_3). HRMS calcd for $C_{17}H_{15}N_3O_3$ $[M+H]^+$: 310.1186, found 310.1180.

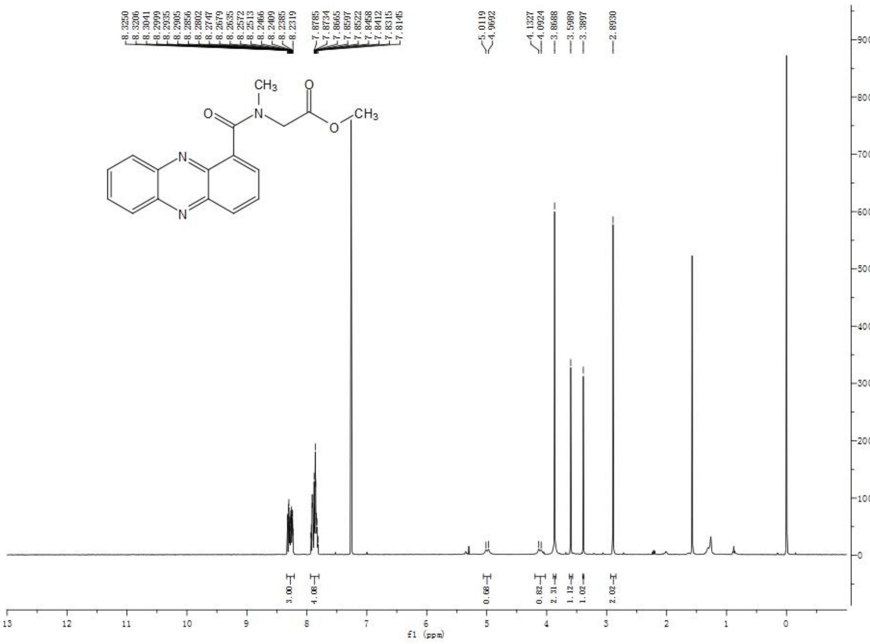

Figure S23. ^{1}H-NMR Spectrum of compound **3g**

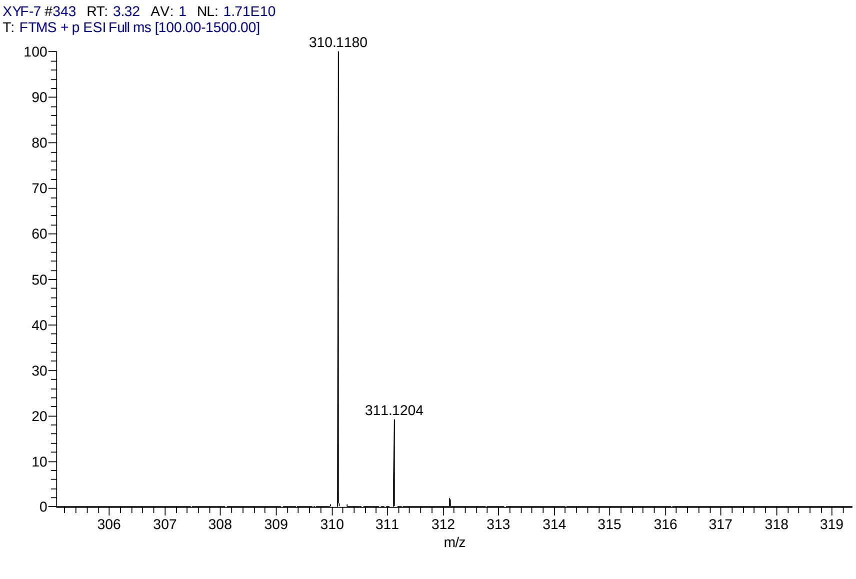

Figure S24. HRMS Spectrum of compound **3g**

Methyl 2-(N-ethylphenazine-1-carboxamido)acetate (3h) :

Yellow solid; yield: 69%; m.p. 121 – 123°C; ^{1}H NMR (400 MHz, $CDCl_3$) δ 8.34 – 8.18 (m, 3.00H, Phenazine-H), 7.93 – 7.80 (m, 4.04H, Phenazine-H), 4.87 (d, *J* = 17.2 Hz, 0.71H, N-CH_2-Methyl), 4.20 – 4.00 (m, 1.05H, N-CH_2), 3.86 (d, *J* = 10.2 Hz, 2.74H, $COOCH_3$), 3.68 (s, 0.31H, $COOCH_3$), 3.57 (s, 1.01H, N-CH_2), 3.24 (dt, *J* = 14.6, 7.2 Hz, 1.39H, N-CH_2-Methyl), 1.42 (t, *J* = 7.2 Hz, 1.00H, N-Methylene-CH_3), 1.04 (t, *J* = 7.2 Hz, 2.09H, N-Methylene-CH_3). HRMS calcd for $C_{18}H_{17}N_3O_3$ $[M+H]^+$: 324.1343, found 324.1338.

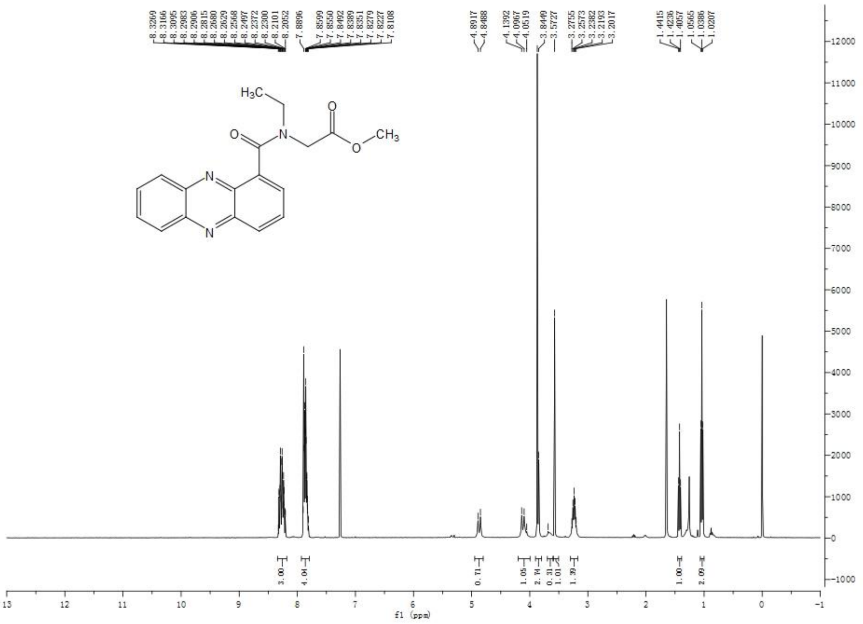

Figure S25. ^{1}H-NMR Spectrum of compound **3h**

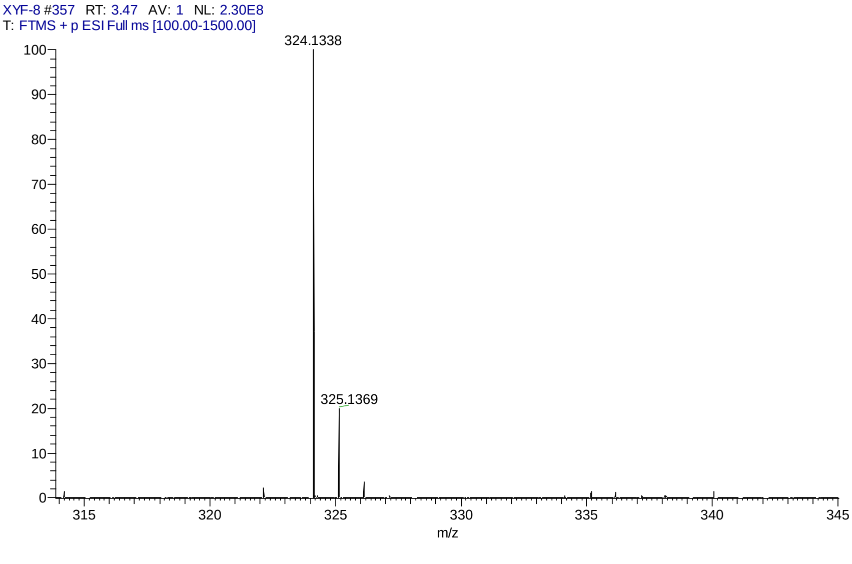

Figure S26. HRMS Spectrum of compound **3h**

Methyl 2-(N-isopropylphenazine-1-carboxamido)acetate (3i) :

Yellow solid; yield: 71%; m.p. 171 – 173°C; ^{1}H NMR (400 MHz, $CDCl_3$) δ 8.37 – 8.15 (m, 3.00H, Phenazine-H), 7.98 – 7.77 (m, 4.00H, Phenazine-H), 5.26 (d, *J* = 6.6 Hz, 0.27H, N-CH), 4.71 – 4.60 (m, 0.83H, N-CH), 4.07 – 3.95 (m, 0.88H, N-CH_2), 3.93 – 3.83 (m, 2.37H, $COOCH_3$), 3.82 – 3.69 (m, 1.27H, N-CH), 3.61 – 3.52 (m, 0.68H, $COOCH_3$), 1.45 (s, 0.74H, 2×CH_3), 1.30 (s, 0.86H, 2×CH_3), 1.14 (dd, *J* = 9.8, 4.6 Hz, 2.07H, 2×CH_3), 1.10 (dd, *J* = 10.0, 4.8 Hz, 2.42H, 2×CH_3) . HRMS calcd for $C_{19}H_{19}N_3O_3$ $[M+H]^+$: 338.1499, found 338.1492.

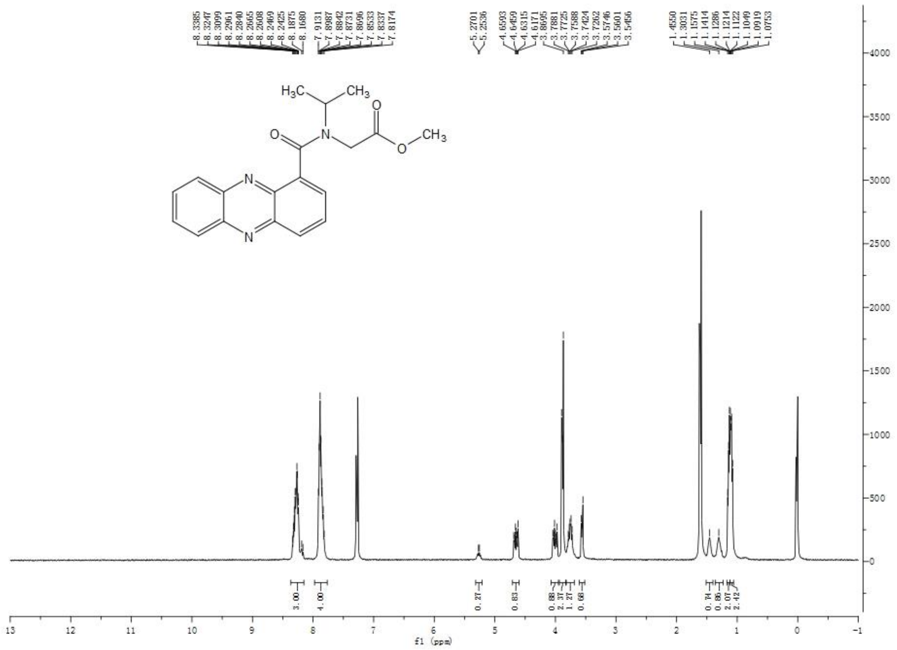

Figure S27. ^{1}H-NMR Spectrum of compound **3i**

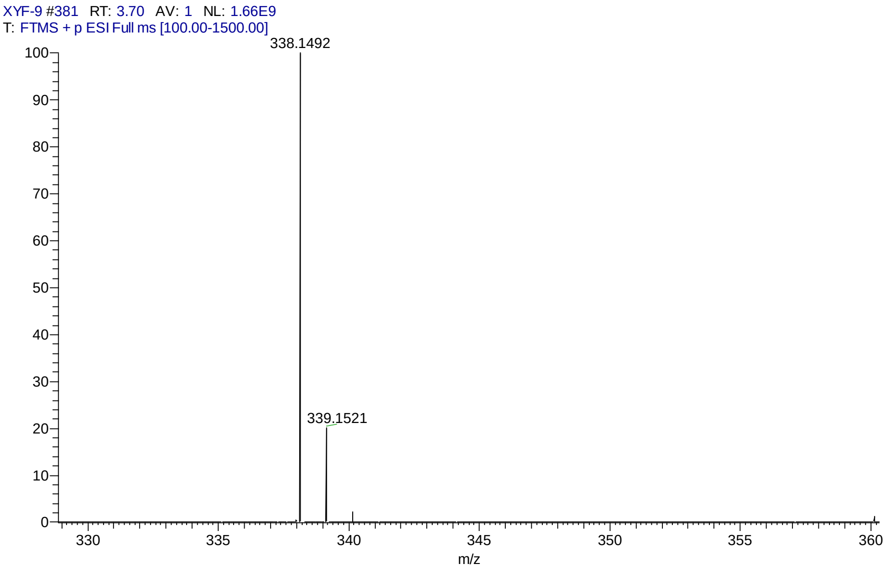

Figure S28. HRMS Spectrum of compound **3i**

Methyl 2-(N-(tert-butyl)phenazine-1-carboxamido)acetate (3j) :

Yellow solid; yield: 81%; m.p. 127 – 129°C; ^{1}H NMR (400 MHz, $CDCl_3$) δ 8.27 – 8.17 (m, 3.00H, Phenazine-H), 7.90 – 7.74 (m, 4.10H, Phenazine-H), 4.05 (d, J = 19.0 Hz, 1.00H, N-CH_2), 3.81 (d, J = 19.0 Hz, 1.03H, N-CH_2), 3.58 (s, 3.04H, $COOCH_3$), 1.71 (s, 8.88H, 3×CH_3). HRMS calcd for $C_{20}H_{21}N_3O_3$ [M+H]$^+$: 352.1656, found 352.1647.

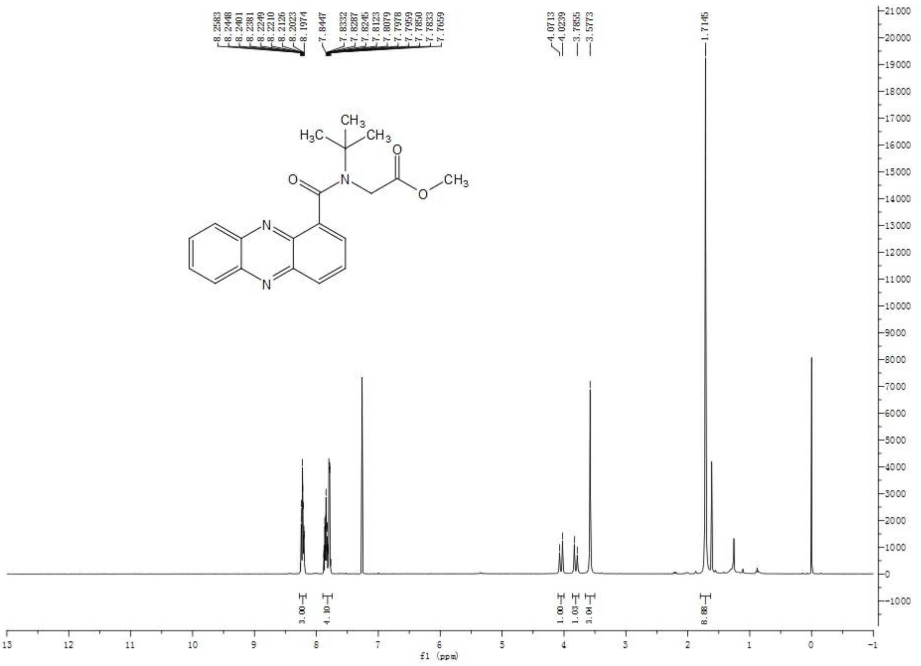

Figure S29. ^{1}H-NMR Spectrum of compound **3j**

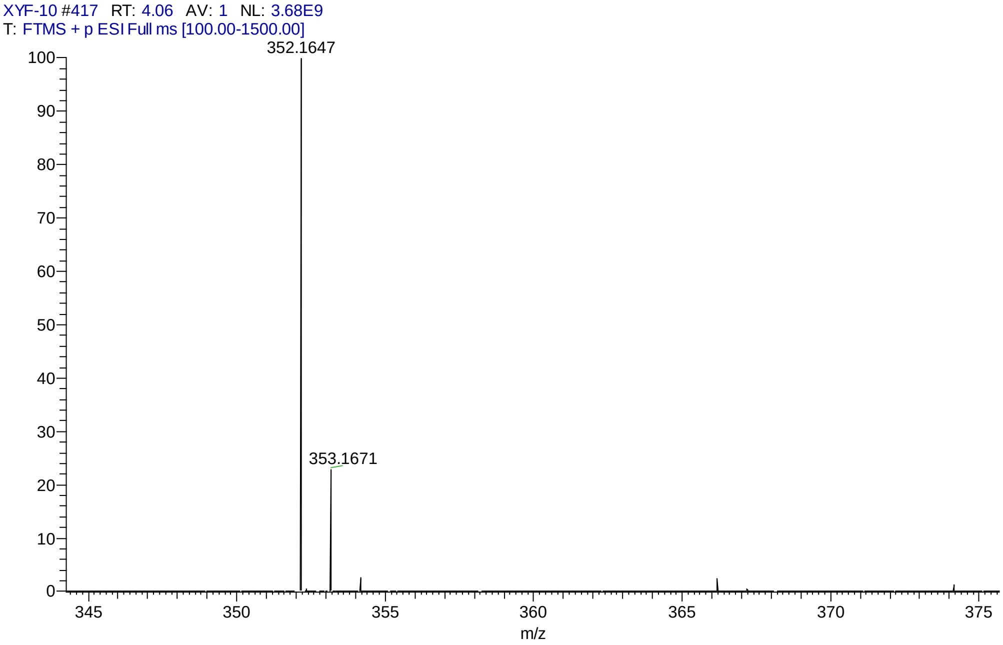

Figure S30. HRMS Spectrum of compound **3j**

Methyl 2-(N-benzylphenazine-1-carboxamido)acetate (3k) :

Yellow solid; yield: 76%; m.p. 182 – 184°C; ^{1}H NMR (400 MHz, $CDCl_3$) δ 8.39 – 8.19 (m, 3.00H, Phenazine-H), 7.98 – 7.82 (m, 3.98H, Phenazine-H), 7.73 (d, J = 7.4 Hz, 0.93H, Benzene-H), 7.49 (t, J = 7.6 Hz, 0.98H, Benzene-H), 7.41 – 7.31 (m, 1.56H, Benzene-H), 7.26 – 7.16 (m, 1.48H, Benzene-H), 5.85 (d, J = 15.6 Hz, 0.51H, Benzene-CH_2), 4.81 (d, J = 17.2 Hz, 0.58H, Benzene-CH_2), 4.55 (d, J = 15.6 Hz, 0.60H, N-CH_2), 4.41 – 4.30 (m, 1.03H, Benzene-CH_2), 3.93 (d, J = 17.2 Hz, 0.59H, N-CH_2), 3.83 (s, 1.60H, $COOCH_3$), 3.73 (d, J = 15.6 Hz, 0.84H, N-CH_2), 3.57 (s, 1.40H, $COOCH_3$). HRMS calcd for $C_{23}H_{19}N_3O_3$ $[M+H]^+$: 386.1499, found 386.1492.

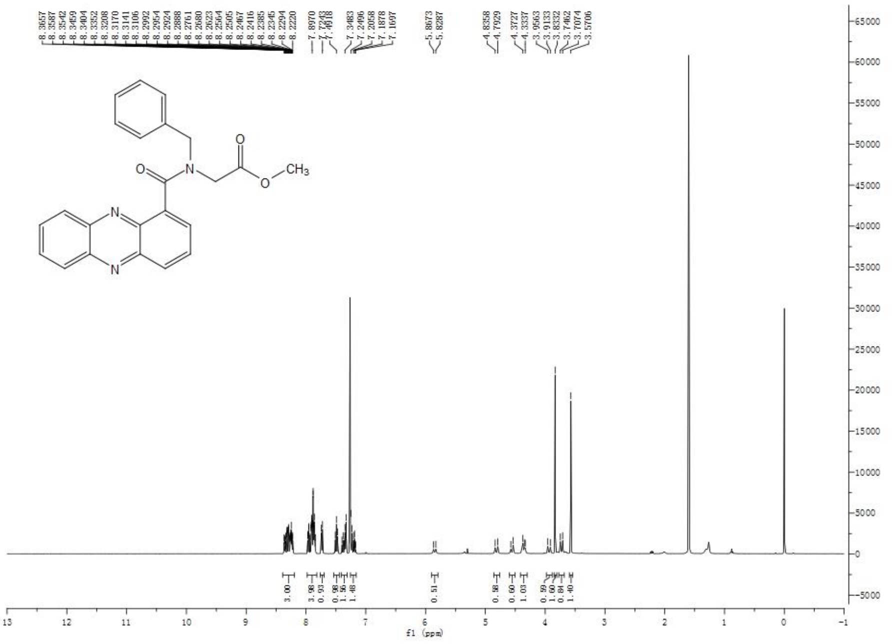

Figure S31. ^{1}H-NMR Spectrum of compound **3k**

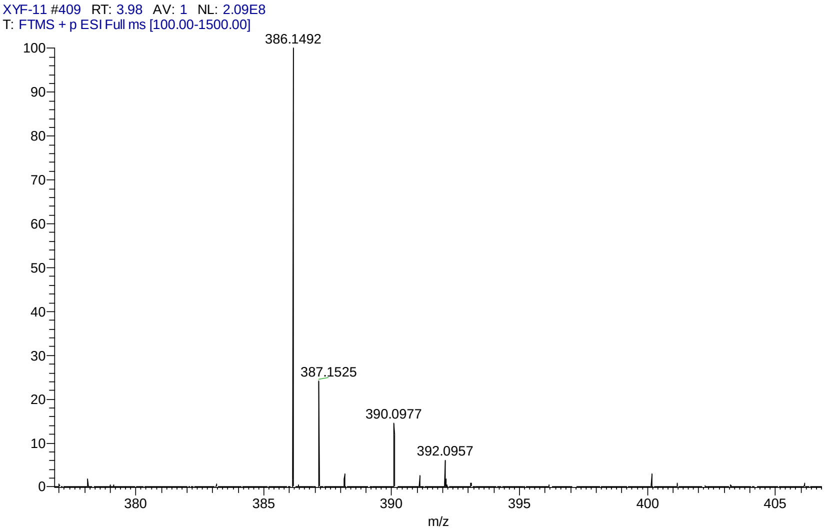

Figure S32. HRMS Spectrum of compound **3k**

Dimethyl 2,2'-((phenazine-1-carbonyl)azanediyl)diacetate (3l) :

Yellow solid; yield: 76%; m.p. 165 – 167°C; ^{1}H NMR (400 MHz, $CDCl_3$) δ 8.36 – 8.21 (m, 3.00H, Phenazine-H), 7.98 – 7.81 (m, 4.07H, Phenazine-H), 4.69 (s, 2.04H, N-CH_2), 3.99 (s, 2.01H, N-CH_2), 3.86 (s, 2.98H, $COOCH_3$), 3.49 (s, 3.00H, $COOCH_3$). HRMS calcd for $C_{19}H_{17}N_3O_5$ [M+H]$^+$: 368.1241, found 368.1234.

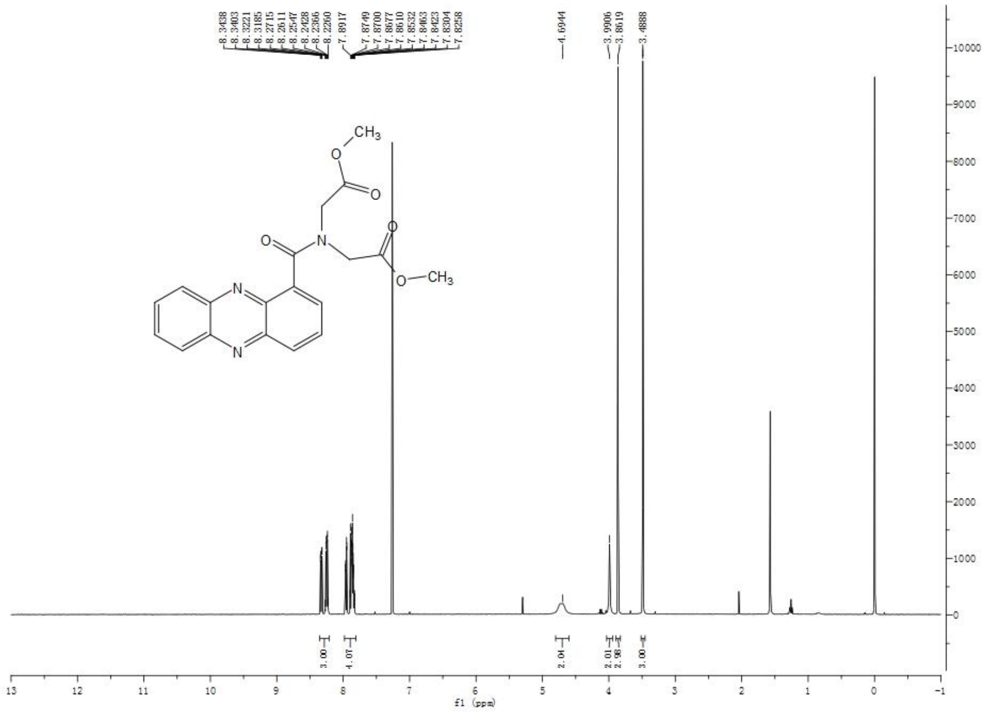

Figure S33. ^{1}H-NMR Spectrum of compound **3l**

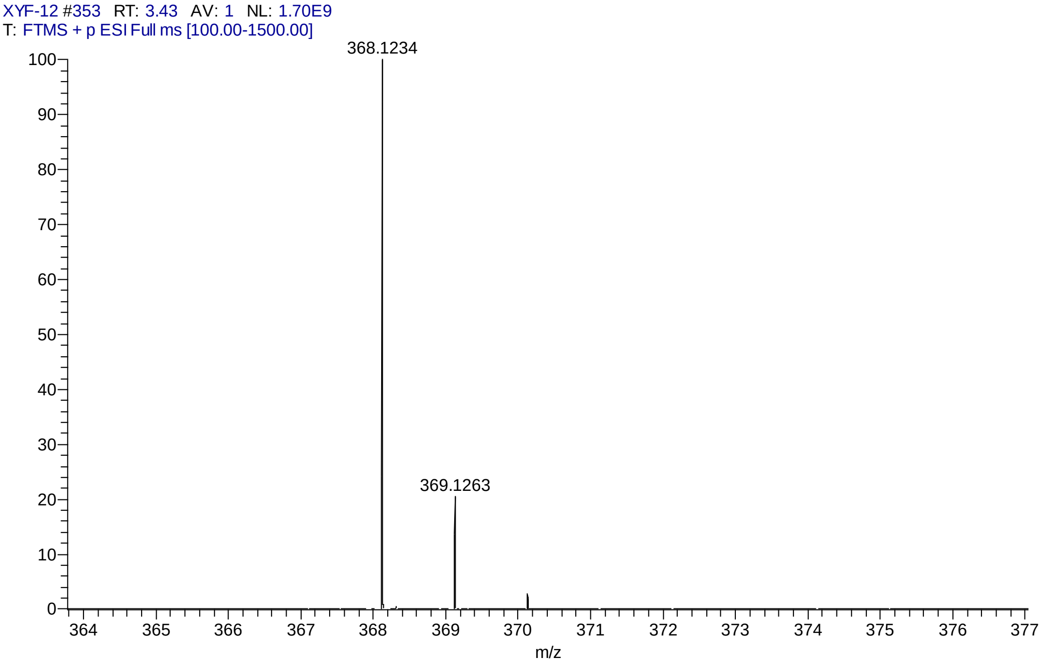

Figure S34. HRMS Spectrum of compound **3l**

2-(N-methylphenazine-1-carboxamido)acetic acid (4a) :

Yellow solid; yield: 90%; m.p. 214 – 215°C; ^{1}H NMR (400 MHz, DMSO-d_6) δ 12.79 (s, 1.02H, COOH), 8.38 – 8.13 (m, 3.00H, Phenazine-H), 8.07 – 7.92 (m, 3.02H, Phenazine-H), 7.86 (dd, J = 6.8, 1.2 Hz, 0.57H, Phenazine-H), 7.78 (dd, J = 6.8, 1.2 Hz, 0.48H, Phenazine-H), 4.66 (d, J = 17.2 Hz, 0.61H, N-CH_2), 4.08 (d, J = 17.2 Hz, 0.62H, N-CH_2), 3.81 (d, J = 11.2 Hz, 0.83H, N-CH_2), 3.20 (s, 1.43H, N-CH_3), 2.77 (s, 1.65H, N-CH_3). HRMS calcd for $C_{16}H_{13}N_3O_3$ $[M+H]^+$: 296.1030, found 296.1024.

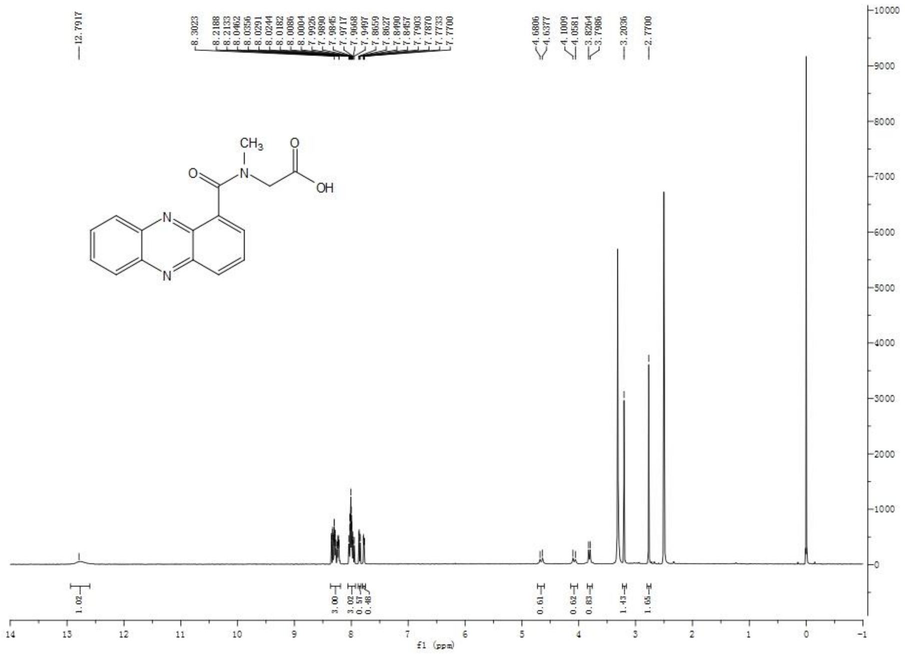

Figure S35. ^{1}H-NMR Spectrum of compound **4a**

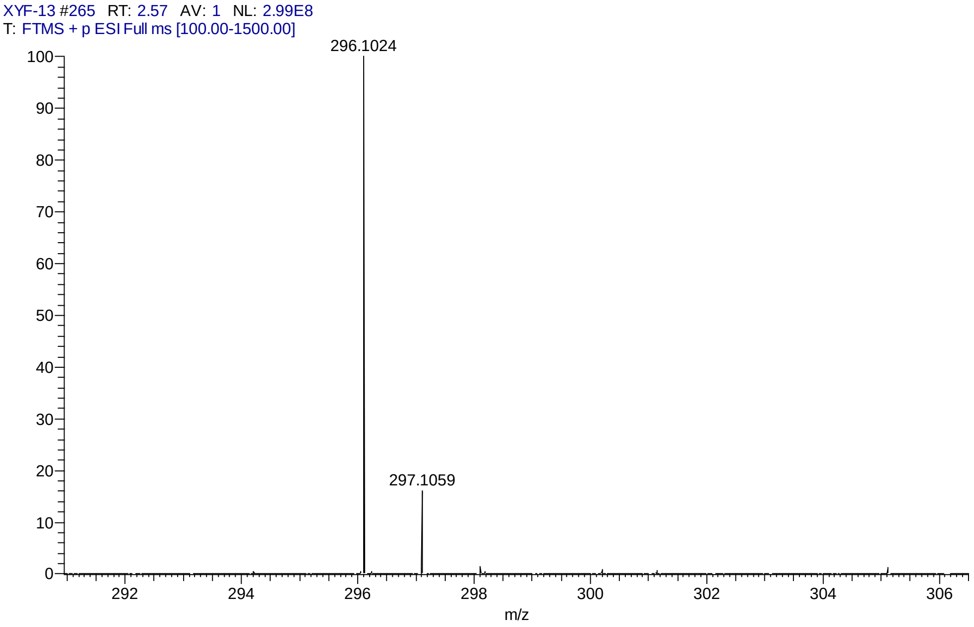

Figure S36. HRMS Spectrum of compound **4a**

2-(N-ethylphenazine-1-carboxamido)acetic acid (4b) :

Yellow solid; yield: 91%; m.p. 119 – 121°C; ^{1}H NMR (400 MHz, DMSO-d_6) δ 12.69 (s, 1.02H, COOH), 8.38 – 8.15 (m, 3.00H, Phenazine-H), 8.07 – 7.75 (m, 4.08H, Phenazine-H), 4.55 (d, J = 17.2 Hz, 0.64H, N-CH_2-Methyl), 4.09 (d, J = 17.0 Hz, 0.64H, N-CH_2), 3.88 (d, J = 18.6 Hz, 0.76H, N-CH_2), 3.70 (d, J = 18.0 Hz, 0.43H, N-CH_2), 3.58 – 3.44 (m, 0.44H, N-CH_2), 3.12 (dt, J = 21.2, 7.2 Hz, 1.39H, N-CH_2-Methyl), 1.31 (t, J = 7.2 Hz, 1.16H, N-Methylene-CH_3), 0.94 (t, J = 7.0 Hz, 1.87H, N-Methylene-CH_3). HRMS calcd for $C_{17}H_{15}N_3O_3$ [M+H]$^+$: 310.1186, found 310.1181.

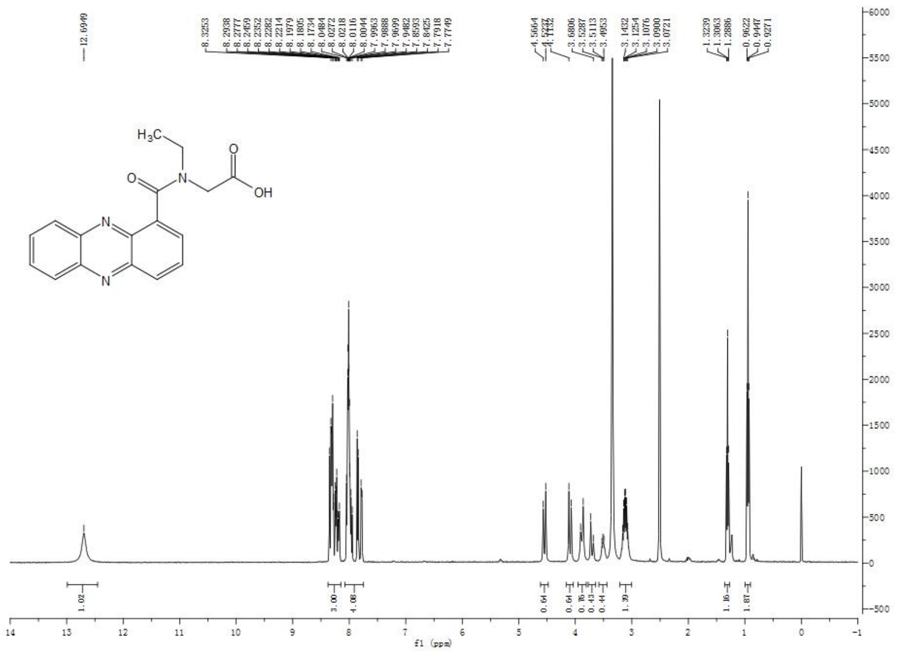

Figure S37. ^{1}H-NMR Spectrum of compound **4b**

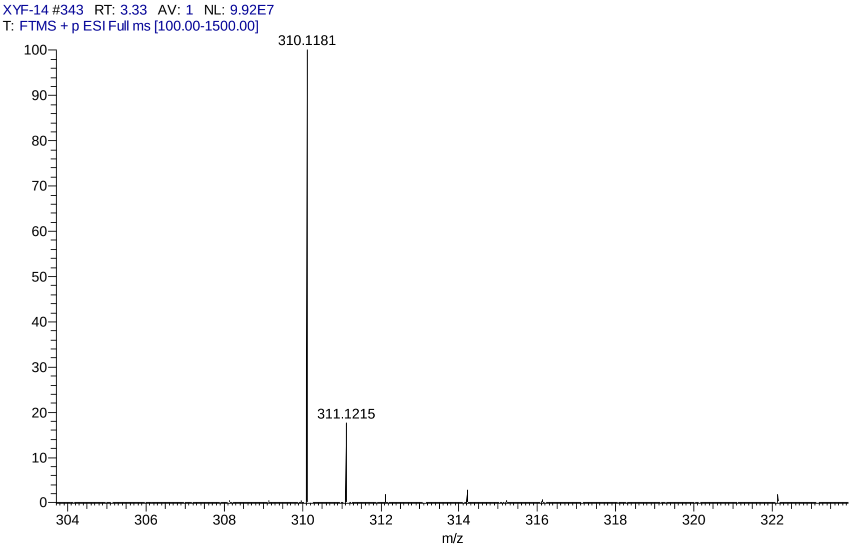

Figure S38. HRMS Spectrum of compound **4b**

2-(N-isopropylphenazine-1-carboxamido)acetic acid (4c) :

Yellow solid; yield: 89%; m.p. 230 – 232°C; ^{1}H NMR (400 MHz, DMSO-d_6) δ 12.57 (s, 1.00H, COOH), 8.36 – 8.10 (m, 2.98H, Phenazine-H), 8.07 – 7.73 (m, 4.00H, Phenazine-H), 4.98 (dt, J = 13.2, 6.4 Hz, 0.30H, N-CH), 4.31 (d, J = 17.0 Hz, 0.78H, N-CH), 4.04 (d, J = 17.0 Hz, 0.80H, N-CH_2), 3.90 (d, J = 18.8 Hz, 0.27H, N-CH_2), 3.58 (dt, J = 13.2, 6.6 Hz, 0.82H, N-CH_2), 3.47 (d, J = 19.2 Hz, 0.29H, N-CH_2), 1.29 (dd, J = 54.2, 6.6 Hz, 1.99H, 2×CH_3), 1.04 (d, J = 6.6 Hz, 2.09H, 2×CH_3), 1.01 (d, J = 6.6 Hz, 2.25H, 2×CH_3) . HRMS calcd for $C_{18}H_{17}N_3O_3$ $[M+H]^+$: 324.1343, found 324.1339.

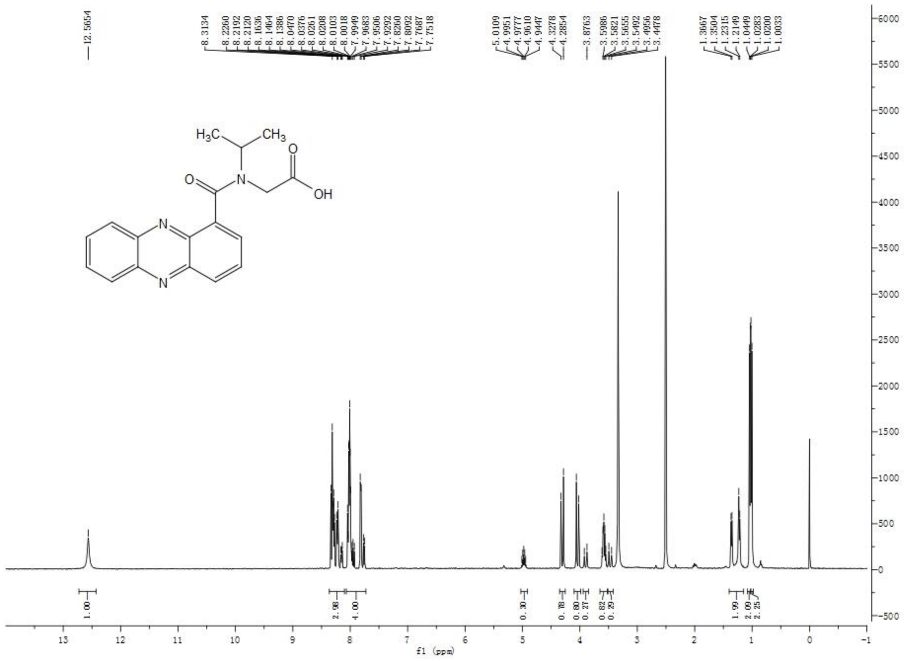

Figure S39. ^{1}H-NMR Spectrum of compound **4c**

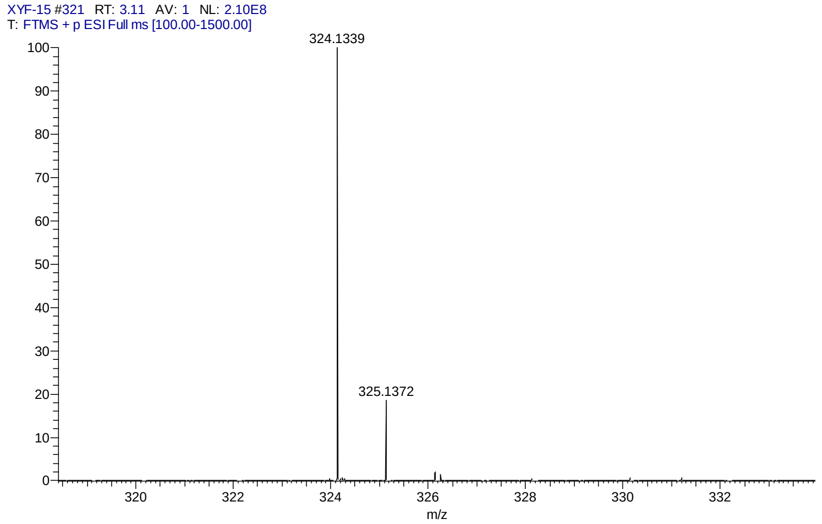

Figure S40. HRMS Spectrum of compound **4c**

2-(N-(tert-butyl)phenazine-1-carboxamido)acetic acid (4d) :

Yellow solid; yield: 92%; m.p. 213 – 214°C; ^{1}H NMR (400 MHz, DMSO-d_6) δ 12.56 (s, 1.02H, COOH), 8.32 – 8.11 (m, 3.00H, Phenazine-H), 8.05 – 7.89 (m, 3.00H, Phenazine-H), 7.70 (d, J = 6.7 Hz, 0.97H, Phenazine-H), 4.05 (d, J = 19.2 Hz, 1.02H, N-CH_2), 3.62 (d, J = 19.2 Hz, 1.02H, N-CH_2), 1.63 (s, 8.66H, 3×CH_3). HRMS calcd for $C_{19}H_{19}N_3O_3$ $[M+H]^+$: 338.1499, found 338.1491.

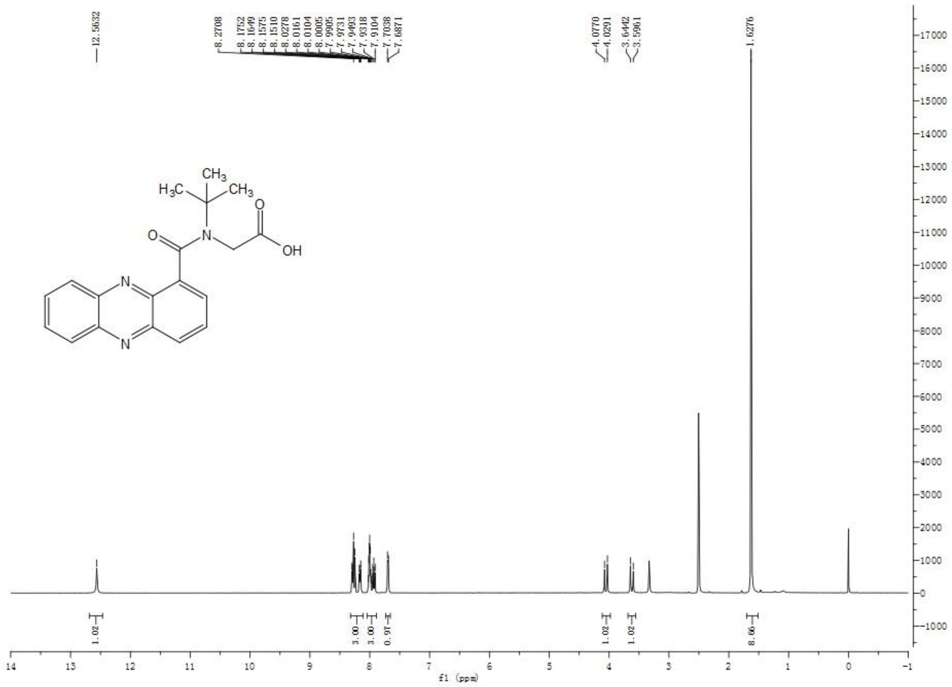

Figure S41. ^{1}H-NMR Spectrum of compound **4d**

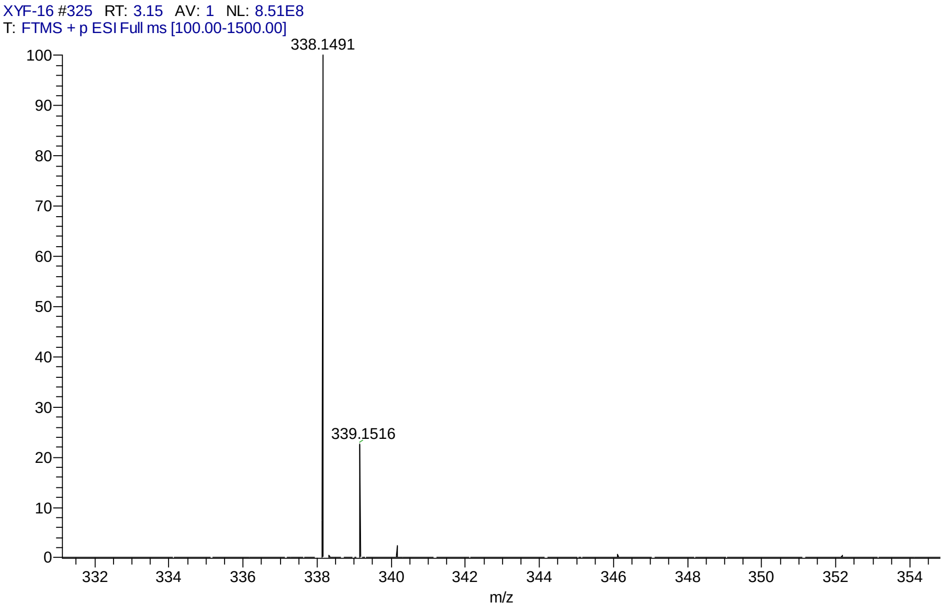

Figure S42. HRMS Spectrum of compound **4d**

2-(N-benzylphenazine-1-carboxamido)acetic acid (4e) :

Yellow solid; yield: 87%; m.p. 152 – 154°C; ^{1}H NMR (400 MHz, DMSO-*d*) δ 12.76 (s, 0.96H, COOH), 8.37 – 8.20 (m, 3.00H, Phenazine-H), 8.11 – 7.95 (m, 3.03H, Phenazine-H), 7.87 (ddd, *J* = 17.8, 6.8, 1.2 Hz, 1.02H, Phenazine-H), 7.77 (d, *J* = 7.2 Hz, 1.25H, Benzene-H), 7.54 (t, *J* = 7.6 Hz, 1.30H, Benzene-H), 7.38 (t, *J* = 7.4 Hz, 0.68H, Benzene-H), 7.29 (d, *J* = 7.4 Hz, 0.72H, Benzene-H), 7.20 (t, *J* = 7.4 Hz, 0.74H, Benzene-H), 7.11 (t, *J* = 7.2 Hz, 0.38H, Benzene-H), 4.57 – 4.21 (m, 2.01H, Benzene-CH$_2$), 3.69 (dt, *J* = 41.2, 17.4 Hz, 2.04H, N-CH$_2$). HRMS calcd for $C_{22}H_{17}N_3O_3$ [M+H]$^+$: 372.1343, found 372.1336.

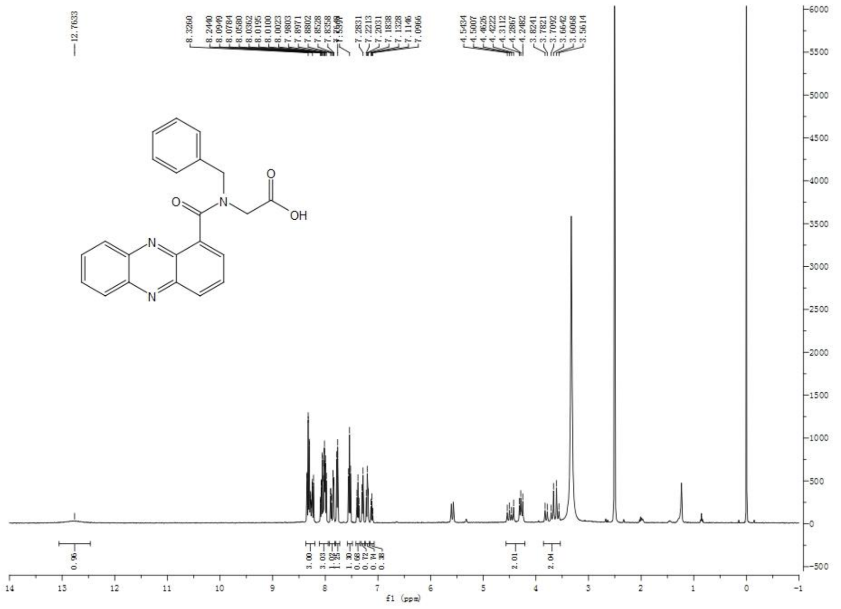

Figure S43. ^{1}H-NMR Spectrum of compound **4e**

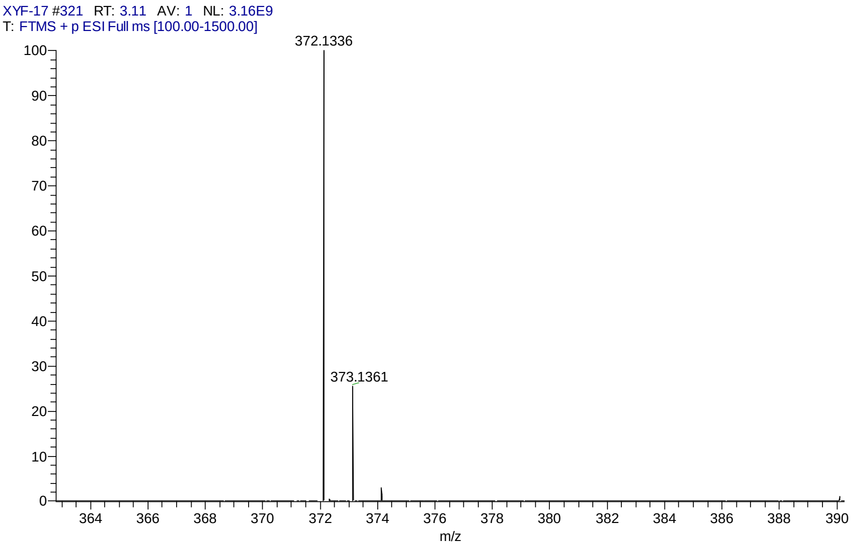

Figure S44. HRMS Spectrum of compound **4e**

2,2'-((phenazine-1-carbonyl)azanediyl)diacetic acid (4f) :

Yellow solid; yield: 90%; m.p. 224 – 226°C; ^{1}H NMR (400 MHz, DMSO-d_6) δ 12.75 (s, 2.02H, COOH), 8.48 – 8.36 (m, 2.03H, Phenazine-H), 8.29 (s, 1.00H, Phenazine-H), 8.16 – 8.04 (m, 3.00H, Phenazine-H), 7.94 (d, J = 5.8 Hz, 1.00H, Phenazine-H), 4.50 (s, 2.01H, N-CH_2), 3.96 (s, 2.03H, N-CH_2). HRMS calcd for $C_{17}H_{13}N_3O_5$ $[M+H]^+$: 340.0928, found 340.0922.

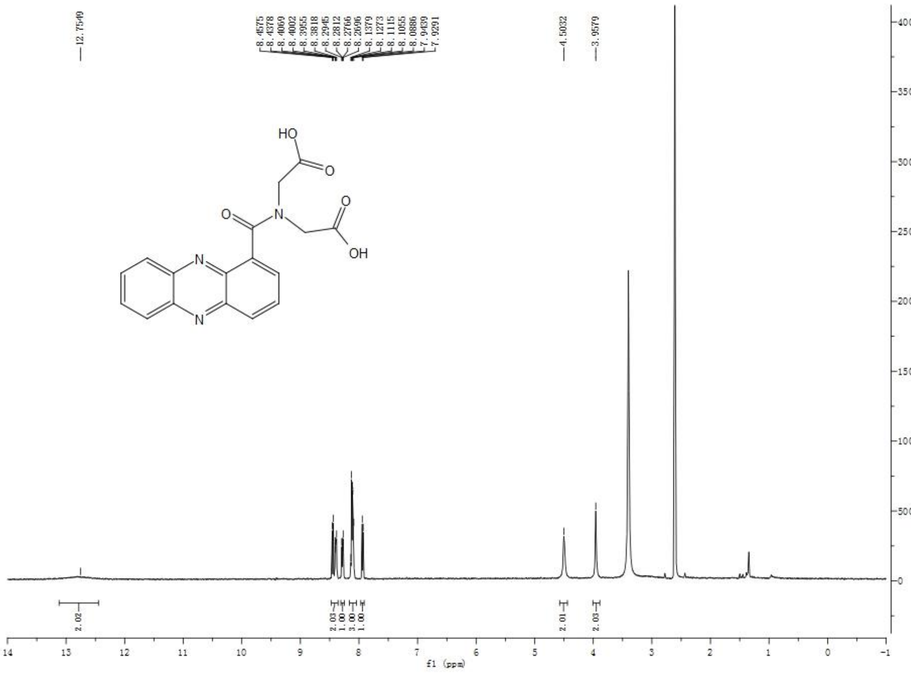

Figure S45. ^{1}H-NMR Spectrum of compound **4f**

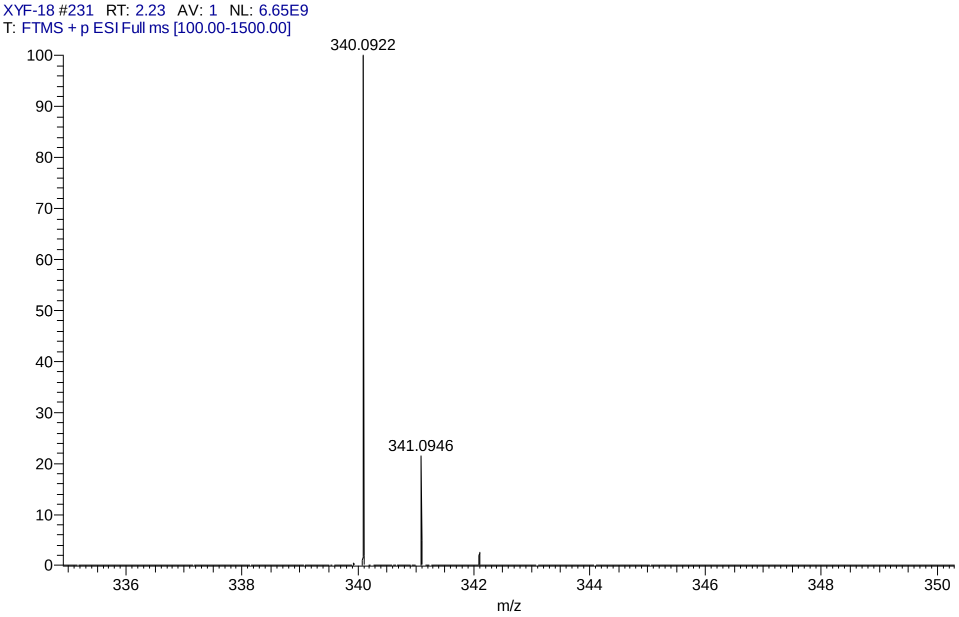

Figure S46. HRMS Spectrum of compound **4f**
